# Climate Events, Food Environment, and Nutritional Outcomes: A Scoping Review

**DOI:** 10.1007/s13668-026-00809-6

**Published:** 2026-10-29

**Authors:** Brenda da Cunha Carvalho, Ariene Silva Carmo, Letícia Lopes Vieira, Raquel Canuto, Dafne Pavão Schattschneider, Tamires Nunes dos Santos, Daniela Canella, Larissa Loures Mendes

**Affiliations:** 1 Department of Preventive Medicine, Faculty of Medicine, Federal University of Minas Gerais, Belo Horizonte, Brazil; 2 Department of Clinical and Social Nutrition, School of Nutrition, Federal University of Ouro Preto, Ouro Preto, Brazil; 3 Department of Nutrition, School of Nursing, Federal University of Minas Gerais, Av. Prof. Alfredo Balena, 190 - Santa Efigênia, Belo Horizonte, MG 30130-100 Brazil; 4 Department of Nutrition, Faculty of Medicine, Federal University of Rio Grande do Sul, Porto Alegre, Brazil; 5 Department of Applied Nutrition, Institute of Nutrition, Rio de Janeiro State University, Rio de Janeiro, Brazil

**Keywords:** Extreme climate events, Climate variability, Nutritional status, Food security, Food environment

## Abstract

**Purpose of Review:**

This scoping review aims to present the scientific literature on the associations of climate events (extreme weather events and climate variability) with anthropometric outcomes, food security, and the food environment. It aims to map evidence, analyze the existing findings, identify gaps, and improve the understanding of how climate events are associated with these outcomes, offering insights into the development of intersectoral actions in the face of the emerging risks of the climate crisis.

**Recent Findings:**

From the 78 included studies (47.4% published after 2021), most were conducted in Africa (50.0%) and focused on children under five years old (82.0%). Child undernutrition, particularly stunting (50.0%), was the most studied outcome. Few studies addressed food security (12.8%) or the food environment (3.8%). Climate variability (rainfall and/or temperature) was the most frequent exposure (71.7%), while 35.8% evaluated extreme weather events. Evidence indicates that precipitation variability – such as rainfall anomalies – are associated with child stunting and increased food insecurity. Furthermore, increases in mean temperature and heatwaves were linked to reductions in children’s weight-for-height, weight-for-age, and birth weight in most studies.

**Summary:**

The literature demonstrates significant associations of climate events with nutritional status, notably the association between climate variability and child undernutrition. However, it lacks intersectional approaches and comprehensive analyses of outcomes like obesity, food insecurity, and the food environment, as well as extreme weather events. More in-depth, context-oriented research is needed to advance knowledge regarding the relationships of these different types of climate events with different outcomes across various contexts, effectively guiding mitigation and food security policies.

**Supplementary Information:**

The online version contains supplementary material available at https://doi.org/10.1007/s13668-026-00809-6.

## Introduction

Climate change poses one of the biggest challenges of the 21st century, with direct impacts on ecosystems, food systems, and human health on multiple scales [1–3]. Although natural variations are part of the climate dynamics of the planet, there is scientific consensus that the intensification of global warming is mainly due to human activities, such as the burning of fossil fuels, deforestation, and unsustainable agricultural practices [4–6].

These climate-related disruptions manifest through both acute extreme weather events—such as severe droughts, floods, tropical cyclones, heatwaves, and wildfires—and multi-temporal climate variability, characterized by seasonal or annual anomalies in rainfall and temperature patterns [7, 8]. While extreme weather events represent sudden, high-magnitude shocks that acutely disrupt infrastructure and food supply, climate variability reflects gradual fluctuations in meteorological regimes that influence long-term agricultural performance and food security [4, 6, 8]. Differentiating these exposures is essential, as their immediate, cumulative, and systemic impacts differentially affect agricultural production, local food environments, and population health, with heightened severity in structurally vulnerable regions [9, 10].

Climate events are linked to worsening food availability, as well as physical and financial accessibility to healthy foods. As a result, there is an increased occurrence of food insecurity and different forms of malnutrition, including undernutrition and overweight [3, 11]. It is estimated that 74% of Latin American and Caribbean countries already face significant losses in agricultural productivity owing to climate change, which is related to rising prices, lower dietary diversity, and deteriorating food environments [11].

In this context, the climate crisis is part of the global syndemic, as described by Swinburn et al. [12], which connects three major contemporary crises: obesity, undernutrition, and climate change. Unsustainable food systems, wealth-concentrating production models, and the growing production and availability of ultra-processed foods are structural factors that amplify these problems and make it difficult to achieve the Sustainable Development Goals (SDGs), particularly SDG 2 – Zero Hunger and Sustainable Agriculture [13].

Although there is an increasing number of studies investigating the multiple connections between climate events, nutritional outcomes, and the food environment, no scoping review to date has comprehensively synthesized the existing body of scientific evidence on these topics. Therefore, this study aims to present a scoping review of the scientific literature on the associations of climate events with anthropometric outcomes, food security, and the food environment. It aims to map evidence, analyze the existing findings, identify gaps, and offer insights into the development of intersectoral actions in the face of the emerging risks of the climate crisis.

## Methods

### Study Design

This is a scoping review, reported according to the checklist and guidelines of the *Preferred Reporting Items for Systematic Reviews and Meta-Analyses for Scoping Reviews (PRISMA-ScR*) to ensure a robust and reproducible process [14] (Online Resource 1).

### Research Question

The research question was designed according to the PCC (population, concept, and context) strategy. For the population, there were no restrictions of age groups or specific characteristics. The concept addressed in this review was the occurrence of climate events and their relationship with anthropometric outcomes, food security, and the food environment. Contextually, no geographical or cultural restrictions were applied.

The guiding question was: “How have extreme weather events and climate variability (rainfall and/or temperature) been related to anthropometric outcomes, food security, and the food environment?”

### Definition of Outcomes of Interest and Exposure Variables

To ensure conceptual uniformity and clarity on eligibility criteria, outcomes and exposure variables of interest were defined and standardized, as shown in Table 1.

**Table 1:** Definition and operationalization of the outcomes and exposure variables of interest of the present scoping review

Outcome and exposure variable of interest	Conceptual definition*	Inclusion criteria
Outcomes of interest		
Anthropometric outcomes	Physical measurements of the body that reflect an energy and nutritional balance. Anthropometric measurements evaluate the size, proportions, and composition of the body. They are used for evaluating the growth, development, and nutritional status of individuals or populations [15].	Use of anthropometric measurements such as body weight, height, circumferences (waist, hip, or arm), skinfolds (triceps, subscapular skinfold, etc.), anthropometric indices^1^ (such as the Body Mass Index (BMI), height-for-age, weight-for-height, and BMI-for-age z-scores, etc.) and anthropometric indicators^2^ [15].
Food security	Condition of physical, social, and economic access to sufficient, safe, and nutritious food [16].	Use of food insecurity scales or indexes, or indicators of access/availability at the family/community level
Food Environment	Consumer interface with the food system that encompasses the accessibility, affordability, convenience, promotion, quality, and sustainability of foods [17]	Discussion about food environment dimensions (formal vs. informal), logistics (transportation and distribution), price, and/or food availability, using sources such as georeferenced secondary data, local audits, and/or categorization of food outlets that sell food in a post-disaster context [17].
Exposure variable of interest		
Climate Variability	Deviations in average temperature and/or precipitation patterns, including averages, extremes, and the timing of weather conditions over seasons or years, excluding discrete and sudden events [8].	Use of continuous climate indicators: Mean/median of climate variables (such as temperature and/or precipitation) and/or deviation/anomalies indicators (e.g., precipitation z-score, standard deviation, monthly or seasonal anomalies, months with water deficit) [8].
Extreme weather events	Occurrence of extreme weather or climate phenomena, rare or very intense, such as severe droughts, floods, cyclones, heat waves, storms, wildfires, and landslides [8].	Use of perceived measures and objective measures of the occurrence of extreme weather events.

Source: Authors

*Conceptual definitions based on FAO (1996), WHO (2006), Downs et al. (2020) and IPCC (2012)

^1^Anthropometric index: combination of anthropometric measurements (e.g. weight and height) or between an anthropometric and a demographic measurement (e.g. age) [WHO, 2006]

^2^Anthropometric indicators: they correspond to the classification that is assigned to an individual as a result of applying a critical value (cutoff point) to an index [15]

### Eligibility Criteria

Original studies published in peer-reviewed journals and indexed in the databases consulted, without date restriction, were included until September 2025. Such studies investigated the relationship of interest of the present review, including the association of climate events with at least one outcome of interest, as shown in Table 1. Only articles published in English, Spanish, and Portuguese were considered. The following papers were excluded: (a) any type of literature review; and (b) other types of publications and documents (e.g. editorials, letters, book chapters, conference proceedings, etc.).

### Search Strategy

The studies were extracted from MEDLINE databases via PubMed, and from Scopus, SciELO and Web of Science, until September 2025.

The descriptors and terms were extracted from DeCS and MeSH, respectively. However, to maximize sensitivity and scope, this scoping review used a hybrid approach that combined controlled descriptors with free keywords, because this is an emerging theme, and one of the central key terms, ‘food environment’, for example, does not have an established standardized descriptor. The inclusion of these free terms ensured the capture of relevant literature that would not have been identified in a search restricted only to indexed terms. The aim of the search was to retrieve articles that addressed at least one of the outcomes of interest (anthropometric outcomes, food insecurity, food environment). The complete search strings and syntax used for all queried databases are detailed in Table S1 (Online Resource 1).

In addition to the database search, an active search was performed by reading the reference lists of the included studies to identify relevant publications that had not been retrieved in the initial search. This is a strategy aligned with the *PRISMA-ScR* guidelines to expand the scope of this review [14]. The studies were organized, evaluated, and selected by using the software programs Rayyan and Microsoft Excel 365 for Windows).

### Selection of Studies

The papers were selected independently by the reviewers BCC and LLV with the aid of the software Rayyan. In the first stage, the titles and abstracts were analyzed to check compliance with the eligibility criteria. In the second stage, the selected articles were read in full. Any disagreements between the reviewers were resolved with the evaluation of a third reviewer (ASC), both when selecting the abstracts and reading the papers in full. Prior to the beginning of the process, reviewers underwent training to standardize screening and ensure greater consistency. The study selection process occurred according to the PRISMA-ScR recommendations [14], and a detailed flowchart is shown in the results section.

### Data Extraction and Analysis

Data were extracted using a standardized Microsoft Excel spreadsheet. The process involved a total of five reviewers. Extraction was performed by two pairs of reviewers (LLV and BCC; TN and DPS). Each pair was responsible for a part of the papers, and the extracted data were checked by another reviewer (ASC) to ensure that information was consistent and complete. The following information was extracted from each study: title of the research paper, authors, year of publication, continent, sample and location where the study was conducted, population group (in the case of studies that evaluated anthropometric outcomes), type of climate event, identification of outcomes that were analyzed (anthropometric outcomes, food security, and the food environment), in addition to the objective of the study, methods, explanatory variables, outcome variables, and main findings.

The findings were presented through a narrative synthesis, as recommended by the PRISMA-ScR guidelines. The extracted variables were described by absolute and relative frequencies to support this synthesis.

The quality of the studies was not evaluated, as expected from a scoping review [14].

## Results

### Search Results and Screening

**Fig. 1:**
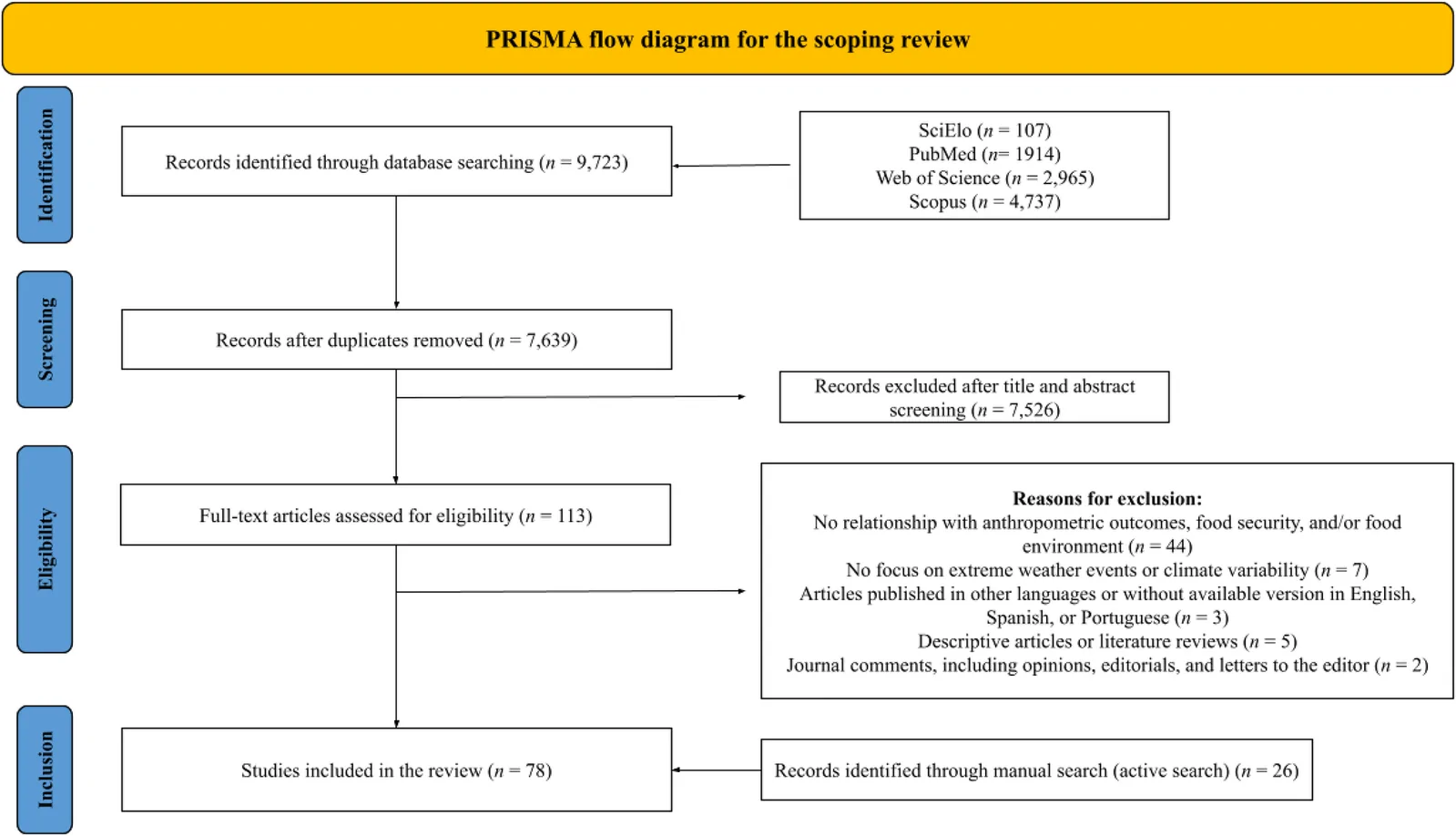
PRISMA flow diagram detailing the systematic search, selection process and exclusion criteria resulting in 78 included studies

The database search identified 9,723 studies, of which 2,084 were duplicates. After the reading of titles and abstracts, 7,526 articles were removed because they did not meet the inclusion criteria. Next, 113 articles were read in full, and 52 of them were considered eligible. Additionally, 26 articles were included from the active search, and a total of 78 papers were included in this review (Fig. 1).

### General Characteristics

Table S2 (Online Resource 1) presents the characteristics of each study included in the review in terms of sample/locality, type of climate event, and outcomes related to anthropometric measurements, food security, and the food environment. These characteristics are summarized in Table 2.

**Table 2:** General characteristics of studies on the relationship between climate events and anthropometric outcomes, food security, and food environment included in the present review (*n* = 78)

Characteristics of the studies	*N*	%
Continent*		
South America	6	7.7
North America	7	9.0
Africa	39	50.0
Asia	29	37.2
Oceania	2	2.6
Europe	1	1.3
Year of publication*		
Up to 2010	2	2.6
2011–2015	12	15.4
2016–2020	27	34.6
2021–2025	37	47.4
Outcomes*		
Height-for-age z-score/ stunting/ severe stunting	39	50.0
Weight-for-age z-score/ underweight/ severe underweight	19	24.3
Weight-for-height z-score/ wasting/ severe wasting/ overweight	29	37.1
Body-mass-index-for-age z-score/ Body Mass Index/ overweight	4	5.1
Birth weight	9	11.5
Other anthropometric outcomes**	13	16.6
Food security	10	12.8
Food environment	3	3.8
Climate events*		
Precipitation	44	56.4
Temperature	38	48.7
Drought	10	12.8
Heat waves/ heat stress	3	3.8
Flood	12	15.4
Aridity	2	2.6
Cyclone, tornado, hurricane, tropical storm	5	6.4
Wildfire	2	2.6
Cold waves	1	1.3
Humidity	2	2.6
Population groups in studies that evaluated anthropometric outcomes*		
Children under 5 years of age	64	82.0
Children between 6 and 10 years old	5	6.4
Adolescents	1	1.3
Adults	4	5.1

Source: Authors

*The categories of these variables are not mutually exclusive; therefore, the sum of the proportions may not correspond to 100%, since the same study can be classified into more than one category of this variable

** Other anthropometric outcomes evaluated: weight gain (*n* = 1, 1.3%); sum of skinfolds (*n* = 1, 1.3%); waist circumference (*n* = 1, 1.3%); arm circumference (*n* = 1, 1.3%); length for gestational age (*n* = 2, 2.6%); weight-for-gestational age (*n* = 2, 2.6%); head circumference for gestational age (*n* = 2, 2.6%); height (*n* = 1, 1.3%); weight (n=%); lean mass (*n* = 1, 1.3%); fat mass (*n* = 1, 1.3%); fetal abdominal circumference (*n* = 1, 1.3%); and fetal femur length (*n* = 1, 1,3%)

**Fig. 2:**
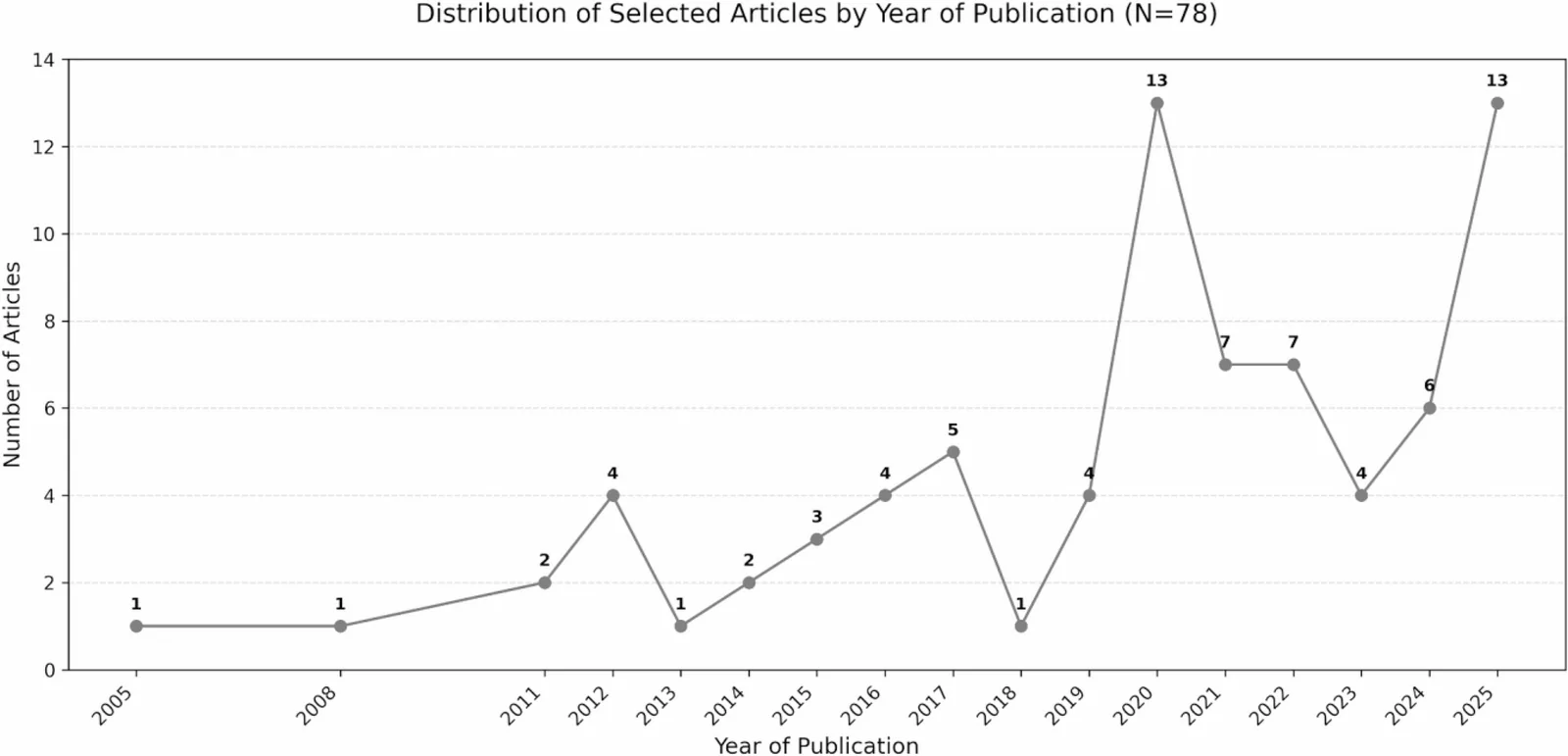
Annual distribution of publications showing the temporal trend and recent increase in research output

Most of the studies were carried out in Africa (50.0%) (Table 2), and there was a higher frequency of studies published between 2021 and 2025 (47.4%) (Table 2; Fig. 2). Regarding outcomes, 67 (85.9%) of the 78 studies evaluated anthropometric outcomes and only 12.8% of the studies (*n* = 10) assessed food security, while the food environment was evaluated as an outcome in only 3 studies (3.8%) (Table 2).

Most of the studies that evaluated anthropometric outcomes (*n* = 64, 82.0%) included samples with children under 5 years of age (Table 2). The most frequent outcome was the standardized anthropometric height-for-age z-score and/or the anthropometric indicator of stunting/severe stunting, which was used by half of the studies. Outcomes related to the anthropometric indicators and indices, namely weight-for-height, weight-for-age, and birth weight, were evaluated in 37.1%, 24.3% and 11.5% of the studies, respectively. Only 7.6% (*n* = 6) of the studies evaluated body fat-related outcomes, such as excess weight according to body mass index (BMI)-for-age or weight-for-height, BMI, waist circumference (WC), and sum of skinfolds.

Most studies evaluated climate variability (*n* = 56, 71.7%) and only 35.8% of the studies (*n* = 28) evaluated associations with at least one type of extreme weather event. Variability in precipitation and temperature was evaluated in 56.4% and 48.7% of the studies, respectively. The most frequent extreme weather events were flooding (15.4%) and drought (12.8%), followed by cyclone, tornado, hurricane, and tropical storm (6.4%) (Table 2).

Table 3 shows the analytical characteristics of the 78 studies included in the review, presenting the outcome variable, the exposure variable, and the main findings of each study.

**Table 3:** Sample, locality, variables, and main findings of studies on the relationship between climate events and anthropometric outcomes, food security, and food environment included in the present review (*n* = 78)

Authors, Year	Sample and location	Explanatory variables	Outcome variables	Main findings
Aguilar & Vicarelli, 2022 [18]	4,111 children from 2 to 6 years of age participating in a longitudinal survey for evaluation of the conditional cash transfer program PROGRESA, with data collected from children born between 1997 and 2000, and a follow-up survey in 2003, in Mexican rural and marginalized communities.	Occurrence of extreme rainfall events during the gestational period or in the children’s first two years of life.	Height-for-age z-score, weight-for-age z-score, stunting	- Children exposed to extreme rainfall during their first two years of life showed a reduction in height-for-age z-scores and in weight-for-age z-scores, and higher odds of stunting. - The impacts were stronger on children affected during in utero growth.
Ahmad et al., 2022 [19]	384 rural households in the Bait community in flood-prone areas of Punjab, Pakistan	Perceived flood-related factors: flooding-induced losses of property, presence of stress caused by flooding, variation in average rainfall between 2010 and 2015, and household income affected by environmental deterioration of floods	Food Security Index	- The perception that flooding-induced losses of property and the presence of stress caused by flooding were inversely associated with worse food insecurity faced by the families. - No statistically significant associations were found between the food security index and other variables of perceived factors.
Ahmed et al., 2021 [20]	9,089 children aged 0 to 59 months living in the households included in the Ethiopia Demographic and Health Survey of 2016	Average daytime land surface temperature, average annual rainfall, aridity, and number of wet days per year.	Prevalence of stunting	- Arid and semi-arid sites were associated with higher odds of stunting in children up to 23 months of age and in children aged 24 to 59 months, without significant interaction for age. - The other climatic factors did not show a statistically significant association with stunting.
Ahsanuzzaman & Islam, 2020 [21]	7,861 children aged 0 to 59 months living in households included in the Bangladesh Demographic and Health Survey of 2011.	Living in areas affected/unaffected by cyclones Sidr and Aila, during in utero and newborn stages or up to 12 months of life.	Height-for-age z-score, weight-for-age z-score, weight-for-height z-score, stunting, underweight, and wasting according to weight-for-height index	- Children exposed to areas affected by cyclones, at both the in utero and newborn stages, had a significant reduction in the height-for-age z-score and low weight-for-age z-score, and they are more likely to be stunted and have low weight-for-age. - In general, there were more significant impacts from the cyclone Sird, which was more severe than Aila. - Most of the results found for the effect of cyclones on the standardized weight-for-height indicator were not statistically significant.
Alterman et al., 2024 [22]	Data on 1,100,576 infants born in Israel between 2011 and 2019 from the National Network of Maternal and Child Health Clinics	Ambient temperature during the prenatal period and one month after birth (in quintiles)	Rapid infant weight gain	- The cumulative adjusted relative risk for the for the highest quintile of prenatal and postnatal exposure to high temperatures was associated with a higher risk of rapid infant weight gain. - The associations with exposure to high temperatures were stronger during early and mid-pregnancy and in the first four postnatal weeks, while lower temperatures at the end of pregnancy were also associated with a higher risk
Alterman et al., 2025 [23]	Infants (968,534 aged 1 year old, and of these, 623,890 evaluated at 2 years of age) born between 2011 and 2019, from the database of the National Network of Maternal and Child Health Clinics, in Israel	Average weekly minimum temperature and average temperature during the first year of life (in quintiles)	Overweight according to the weight-for-length z-score at 1 year of age and according to the BMI-for-age at 2 years of age.	- Infants exposed to the highest average and minimum temperature quintile presented a higher risk of overweight at one year and at two years of age, compared to those exposed to the lowest temperature quintile. - The association was found to be non-linear, with an increase in risk with the increase in temperature, and the risk was stable above 20 °C.
Amegbor et al., 2025 [24]	343,199 under-five-year-old children from 30 countries in Sub-Saharan Africa included in the Demographic and Health Survey (2004–2021)	Standardized precipitation and temperature anomalies (mean and maximum) in the prenatal and postnatal stages (first year of life)	Stunting	- Prenatal and postnatal maximum temperature anomaly was associated with higher odds of stunting while prenatal mean temperature anomaly and prenatal and postnatal precipitation anomaly were associated with lower odds of stunting. - There were nonlinear effects of temperature anomalies on stunting, indicating increased risks in extreme deviations. - Children living in the tropical monsoon zone had lower odds of stunting while certain temperate zones were associated with an increase in stunting.
Ampofo et al., 2025 [25]	Australian adults participating in the longitudinal survey Household, Income and Labour Dynamics in Australia in the waves between 2006 and 2022	Number of days with average temperature > 30 °C and number of days between 2006 and 2022 of exposure to an average temperature below 5 °C and above 30 °C	Body mass index and presence of obesity	- Exposure to a greater number of days with temperatures above 30 °C is associated with a small increase in body mass index and higher odds of obesity. These associations were significant between men and women and more evident among people aged 45 to 64 years. - The mediation analysis identified that the reduction of physical activity and the ‘internal locus of control’ accounts for a significant portion of the total effect of exposure.
Baez et al., 2024 [26]	27,721 under-five-year-old children living in rural households included in demographic and health surveys in the five Southern African countries (Malawi, Tanzania, Mozambique, Zambia, and Zimbabwe)	Occurrence of drought before birth and occurrence of drought in the next season, following birth	Height-for-age z-score	- The occurrence of drought (both prenatal and in the subsequent season) was associated with a reduction in height-for-age z-scores. - Adverse impacts were more intense among children in families with lower purchasing power, characterized by lower maternal education level and houses with primitive roofs.
Baffour et al., 2023 [27]	Cluster-level data of under-five-year-old living in households included in the Bangladesh and Ghana Demographic and Health Survey in 2017-18	Aridity, mean monthly near surface temperature, and mean annual precipitation	Prevalence of stunting	- Higher aridity index values were associated with higher prevalence of stunting in Bangladesh and lower prevalence rates in Ghana. - Mean annual temperature was negatively associated with stunting only in Bangladesh, with no significant effect in Ghana. - Higher mean annual precipitation was associated with higher prevalence of stunting in Ghana alone.
Baker & Anttila-Hughes, 2020 [28]	About 190,000 children aged 1 to 5, living in 28 countries in Sub-Saharan Africa, participating in demographic and health surveys conducted between 1993 and 2012	Average monthly temperature for: the survey month, the 12 months leading up to survey, and the child’s entire lifetime, and during the specific agricultural growing season (corn, wheat, rice).	Weight-for-height, weight-for-age, and height-for-age z-scores	- A significant non-linear relationship was found between high temperatures and reductions in the weight-for-age and weight-for-height indicators. - Temperatures above 25 °C were associated with reductions in weight-for-height, and the effect was most evident in rural areas. - High temperatures during the agricultural growing season were associated with severe losses of weight-for-height, especially in maize and wheat producing regions; there was no effect of temperatures on height-for-age.
Basu et al., 2018 [29]	43,629 full-term infants with low birth weight and 2,032,601 full-term infants with normal birth weight included in the birth records from California’s Center for Health Statistics and Informatics Birth Data File between 1999 and 2013.	Average weekly apparent temperature during full pregnancy, trimesters of pregnancy, first month of pregnancy, month before delivery, and two weeks before delivery.	Low birth weight	- The highest apparent temperature throughout pregnancy, in the second and third trimesters, and in the last two weeks, was associated with low birth weight. - The highest association for low birth weight was observed in the third trimester (above 60ºF). - Mothers who were black, older, delivered male infants, or gave birth during the warm season, showed the highest risks of low birth weight.
Blom et al., 2022 [30]	32,036 children aged 3 to 36 months included in demographic and health surveys conducted between 1993 and 2014 in five West African countries (Benin, Burkina Faso, Ivory Coast, Ghana, and Togo).	Average number of hours per month in different temperature ranges (> 35 °C; 30–35 °C; 25–30 °C; ≤25 °C), both for lifetime exposure (for height-for-age z-scores) and in the last 90 days (for weight-for-height z-scores).	height-for-age z-score, weight-for-height z-score, stunting, and wasting according to the weight-for-height z-score.	- Lifetime exposure to temperatures above 35 °C significantly reduced height-for-age z-score and stunting. - Exposure in the last 90 days to temperatures between 30–35 °C reduced weight-for-height z-score and wasting according to this score. - Projections indicate that a permanent increase of 2 °C in the average temperature would result in an increase of 7.4% in the stunting rate and 6.2% in the wasting rate.
Bonell et al., 2024 [31]	668 children up to two years of age participating in a randomized clinical trial conducted between 2010 and 2015 in West Kiang, Gambia	Universal Thermal Climate Index (composed of heat, humidity, wind speed, and solar radiation) in each trimester of pregnancy	Length-for-gestational age, weight-for-gestational age, and head circumference-for-gestational age z-score for children at birth; weight-for-height, weight-for-age, and height-for-age z-scores for children up to two years of age	- The increase of 1 °C in the daily average Universal Thermal Climate Index in the 1st and 3rd trimesters was associated, respectively, with reduction in weight-for-gestational age and increase in head circumference. - Negative association found between Universal Thermal Climate Index and weight-for-height and weight-for-age Z-scores, with infants from 6 to 18 months being the most affected. - For most exposures, there was no significant association with height-for-age.
Bonjean et al., 2012 [32]	12,571 under-five-year-old children living in rural areas of Burkina Faso, included in the 2008 National Survey on Food Insecurity and Malnutrition.	Annual rainfall variation and positive and negative precipitation shocks during the in utero period and in first months of life, and in the first and second year of life.	Height-for-age z-score	- Greater variation in annual rainfall and positive rainfall shock were associated with higher height-for-age Z-scores. - Negative rainfall shocks were associated with lower height-for-age Z-scores - The impact was significant only for exposure during pregnancy and in the first months of life; there was no significant effect if exposure occurred afterwards. - Boys and families with lower income or lower access to drinking water were more vulnerable to the effects of rainfall.
Cebolla-Boado et al., 2025 [33]	1,316,717 births registered in Spain between 2018 and 2021	Average daily temperature for different gestational weeks, in addition to the number of days with temperatures above 30 °C	Birth weight	- Heat exposure in the 1st trimester of pregnancy resulted in a reduction of approximately 3 g in birth weight exclusively for babies born to immigrant mothers. - Weight loss was more pronounced in the 2nd trimester: about 3 g/day and about 5 g/day for babies born to native mothers and immigrant mothers, respectively. The third trimester showed moderate and similar reductions between the groups. - The risk of low birth weight was increased by exposure in the 1st trimester for immigrants, and in the 2nd trimester for both groups.
Connors et al., 2022 [34]	162 food distribution facilities (food pantries) in census areas of Harris County, Texas, USA	Normal scenario (no flooding) and disruption scenario (after flooding)	Spatial accessibility to food pantries (travel time or distance to the nearest location of each census area, considering road networks)	Much of Harris County has only small increases in travel distance to food pantries due to extreme flooding, but some areas may be left without access to food pantries, including areas where many families live in poverty and areas with high likelihood of food insecurity.
Cooper et al., 2019 [35]	584,662 children aged 0–59 months living in households located mostly in agricultural regions of 127 demographic and health surveys conducted in 53 developing countries over 26 years, from 1990 to 2016.	Standardized Precipitation–Evapotranspiration Index (SPEI), considering 12-, 24- and 36-month intervals, as well as the duration of each child’s lifetime, including time in utero.	Height-for-age z-score	- Periods of drought (SPEI < − 0.4) were associated with a significant reduction in height-for-age scores. Extreme rainfall (SPEI > 1.4) also had a negative impact, but it was less prevalent. - Factors such as nutritional diversity of agricultural systems, higher imports and production of basic crops, higher percentage of irrigated agriculture, political stability, and higher average annual rainfall had a mitigating effect of drought. - Factors such as higher population densities and higher average monthly temperature exacerbated the effects of drought.
Cooper et al., 2019 [36]	1,346 children from Ghana and 3,271 children from Bangladesh under the age of 5 and living in households located in rural areas included in the Feed the Future program conducted in 2012 (Ghana) and 2011 and 2015 (Bangladesh).	Standardized Precipitation–Evapotranspiration Index, considering 12-, 24- and 36-month intervals, as well as the duration of each child’s lifetime, including time in utero.	Height-for-age and weight-for-height z-scores and level of household food insecurity (score).	- Higher levels of mean precipitation in Ghana (at 12 months) were associated with lower weight-for-height (Z-score), while precipitation (at 36 months) was associated with lower height-for-age. - In the arid region of Ghana, the increase in rainfall (long-term and annual patterns) improves food security, and it was associated with less hunger. - In Bangladesh, there was no association between precipitation and height-for-age, while the increase in rainfall (at 48 months) was associated with the higher level of food insecurity.
Danysh et al., 2014 [37]	2,095 children from 7 to 18 years old living in 59 rural villages in Tumbes, Peru.	Two birth date indicators to evaluate the effect of being born before and after the beginning of flooding during the 1997–1998 El Niño, respectively.	Height-for-age z-score, lean mass (kg), and fat mass (kg)	- Height-for-age improved between 1991 and 1997 (before El Niño), but this rate fell during and shortly after the flood event. - Height shortfalls were greater in children from households most likely to be flooded after El Niño. - Children born after the beginning of El Niño presented lower lean mass, while fat mass was not affected.
Datar et al., 2013 [38]	More than 59,000 under-five-year-old children, living in rural areas, included in the Indian National Family and Health Survey conducted in1992-93, 1998-99, and 2005-06.	Exposure to any disaster (drought, flooding, storms, landslide, and earthquakes) in the previous year	Height-for-age z-score, weight-for-age z-score, weight-for-height; stunting, underweight, and wasting according to the weight-for height index	- Exposure to any disaster in the previous year associated with a reduction in height-for-age, weight-for-age, and weight-for-height Z-scores was also associated with higher odds of low height-for-age and low weight-for-age. - The adverse effects on growth results are much lower among boys, younger children (especially under one year of age) and in situations of more educated mothers.
Davenport et al., 2017 [39]	Under-five-year-old children living in households included in the Demographic and Health Survey of thirteen of the poorest sub-Saharan African countries	Average rainfall and number of days in each trimester in which the maximum daytime temperature exceeds 37.7 °C one year before childbirth.	Birth weight and height-for-age z-score	- Temperatures above 37.7 °C in the 1st and 2nd trimesters one year before childbirth are associated with low birth weight. The increase in rainfall is associated with the increase in the height-for-age Z-score. - Changes in heat/drought-induced birth weight are not mitigated by socioeconomic trends, but negative results for height-for-age could be mitigated by these trends. - Results are heterogeneous among countries. In Senegal, higher maternal education and better access to electricity act as mitigating factors, resulting in better health outcomes.
Dell Ninno & Lundberg, 2005 [40]	Data of children up to 60 months of age living in households included in a three-round household survey conducted in Bangladesh, collected between November 1998 and November 1999.	Flood exposure	Height-for-age z-score, weight-for-height z-score, and height	- Flood-exposed children presented stunting and lower overall height compared to children not exposed; this standard was maintained in all three rounds of the survey. - The impact on height was evident in the second year of life (there was no difference in height among infants). - Flood-exposed children did not recover after the end of flooding, as they did not grow faster than children that were not exposed. - Ex ante government programs were more effective than ex post interventions to protect the health of children from the impact of the flood. - There was no significant effect of flood exposure on weight-for-height.
Demeke et al., 2011 [41]	1,477 rural households in Ethiopia, included in the three rounds of the Ethiopian Rural Household Survey dataset (1994, 1999, and 2004)	Satisfaction with rainfall index, annual average rainfall, and rainfall deviation from historical average	Relative food security index (continuous) and relative food security status of households (categorical).	- Satisfaction with rainfall and highest average rainfall in the rainy season are associated with improved food security over time. - Rainfall variability is negatively associated with food security. - Families experiencing a lower rainfall rate and greater rainfall variability are more likely to remain in a persistent state of food insecurity and vulnerability.
Dimitrova & Muttarak, 2020 [42]	220,823 children under five years of age included in the Demographic and Health Surveys of India (2015–2016)	Rainfall anomalies from the in-utero period until the time of the survey	Height-for-age z-score, stunting, and severe stunting	- Cumulative exposure to rainfall anomalies (from the in-utero period until the time of the survey) was associated with a reduction in height-for age Z-scores, and higher odds of stunting and severe stunting. - Maternal education was a protective factor against the adverse impacts of rainfall shocks.
Dimitrova, Bora, 2020 [43]	256,244 children aged 0 to 59 months living in households participating in the Demographic and Health Survey 2015–2016 conducted in India	Standardized precipitation and evapotranspiration index, drought events, and excessive rainfall events, during the period in utero and during the child’s first year of life	Stunting and severe stunting, height-for-age z-score, wasting and severe wasting according to the weight-for-height index and the weight-for-height z-score	- Wet monsoon seasons and excessive rainfall during pregnancy and childhood were associated with a reduction in height-for-age z-score and increased likelihood of stunting. - Increased risk of stunting due to excessive rainfall in households without access to safe sanitation facility, with the occurrence of diarrheal infections (associated with excessive rain) suggested as a mechanism. - Events of drought and excessive rainfall (extremes) were associated with higher values of weight-for-height z-score and lower likelihood of wasting.
Dimitrova, 2021 [44]	21,551 under-five-year-old children living in households included in the Demographic and Health Surveys conducted in 2005, 2011, and 2016 in Ethiopia.	Occurrence of drought	Stunting and wasting according to weight-for-height z-score	- Drought exposure during the main rainy season (meher) was associated with lower risk of stunting in small children. The non-linear model explained the variation in stunting. - The protective effect against stunting was more pronounced in subsistence agricultural regions and in boys. - There was no significant association between seasonal droughts and wasting.
Drysdale et al., 2020 [3]	376 households in Round 1 and 370 households in Round 2 included in household surveys of the iLembeda KwaDuKuza Municipal District, South Africa.	Average annual rainfall of the subdistrict	Food insecurity (score)	- There was a positive association between increased rainfall and food insecurity, whose intensity decreased during the second round of data collection.
Epstein et al., 2019 [45]	3,223 children between 6 and 59 months included in the Uganda National Panel Survey of 2009–2012	Perception about the occurrence of erratic droughts/rainfall and cumulative rainfall deviation in the last 12 months.	Weight-for-age z-score, height-for-age z-score, weight-for-height z-score, underweight, stunting, and wasting according to the weight-for-height index	- Family perception of drought was associated with a higher risk of underweight. - Positive rainfall deviations were associated with a lower risk of underweight and wasting. - There were no significant associations between perceived/objective variables for rainfall and stunting (the results remained with continuous nutrition indicators). - There was a non-linear relationship between rain and wasting: the risk was higher with extreme rainfall reductions, stabilizing after approximately 100 mm below average.
Grace et al., 2012 [46]	2,255 children from 1 to 5 years of age from Kenya residents of the households evaluated in the Kenya Demographic and Health Survey in 2008.	Rainfall levels, variation in rainfall levels, surface temperature, and variation in temperature.	Height-for-age z-score	- There was a positive relationship between rainfall levels and height-for-age z-scores. - There was no association between height-for-age z-scores and temperature or variation in rainfall and temperature.
Grace et al., 2015 [47]	67,769 newborns living in households included in the Demographic and Health Survey of 19 African countries (1986–2010).	Average rainfall by trimester (from year before the child’s birth), average rainfall during the growing season, count of ‘hot’ days per trimester with maximum temperature > 38.7 °C (100 °F), count of ‘hot’ days during the growing season	Birth weight and low birth weight	- Positive relationship between birth weight and precipitation in the growing season, and negative relationship between birth weight and hot days. - Precipitation in all trimesters was associated with lower odds of low birth weight, while hot days (> 100ºF) were associated with an increase in this likelihood. - The combined impact of low precipitation and many hot days during the in utero period specifically increases the risk of low birth weight. This impact is more significant than the effects alone.
Grace et al., 2021 [48]	9,542 newborns living in households included in the Malian Demographic and Health Survey (2000, 2006, and 2012).	Number of days with temperature > 100 °F in each trimester of pregnancy, average monthly precipitation in each trimester of pregnancy, and average monthly maximum temperature in each trimester of pregnancy.	Birth weight and low birth weight	- Exposure to more days with temperature above 100 °F (38 °C) in the 1st and 3rd trimesters, and to higher mean maximum temperature in all trimesters, were associated with low birth weight. - The relationship between average rainfall and birth weight varied in direction and magnitude by trimester.
Hagos et al., 2014 [49]	145 under-five-year-old children from 43 administrative zones in Ethiopia participating in the Ethiopian Demographic and Health Surveys conducted between 1996 and 2004.	Average temperature and average rainfall anomaly during the growing season (June to September)	Severe stunting/stunting, severe underweight/underweight, severe wasting, and wasting according to the weight-for-height index	- The increase in average temperature was associated with lower odds of severe stunting/stunting and severe underweight/underweight. - The increase in rainfall anomaly was associated with higher odds of severe stunting/stunting and severe underweight/underweight. - There was no significant association between climate variables and weight-for-height indicators, except for a positive association of the quadratic term of rainfall with increased wasting and severe wasting. - There was no association between the quadratic terms of rainfall and the height-for-age or weight-for-age indicators.
Hanifi et al., 2022 [50]	19,357 children under the age of 3 born between January 2011 and March 2020 living in the households assessed in the Demographic and Health Surveillance System in 49 villages in the sub-region of Chakaria, Bangladesh	Average monthly temperature, average monthly rainfall, and average monthly relative humidity in the month and year of conception and during the in utero period	Average arm circumference	- Moderately high relative humidity in the month of birth showed a significant positive effect (with nonlinear effects), while rainfall and temperature were not significant. - During pregnancy, high temperatures (> 30 °C), especially in the 3rd trimester, reduced arm circumference; rainfall had negative effects in the 1st and 2nd trimesters. - Exposure to lower temperatures (15–20 °C) in the 2nd trimester of pregnancy was associated with higher arm circumference values. - Variation in humidity during the in utero period did not show a statistically significant association with arm circumference.
Hirvonen et al., 2020 [51]	Children aged 6 to 59 months from Ethiopia, included in two rounds of the Ethiopian Socioeconomic Survey (ESS) (*n* = 1,874 in 2014 and *n* = 1,708 in 2016)	Drought exposure	Height-for-age z-score, weight-for-height z-score, stunting, and wasting according to weight-for-height index	- The drought of 2015 did not impact height-for age and weight-for-height z-scores, nor the categorized measures of these indicators.
Homma et al., 2025 [52]	5,489 under-five-year-old children in rural families from Bangladesh, included in the Bangladesh Integrated Household Survey conducted in three waves: 2011/2012, 2015, and 2018/2019.	Rainfall and temperature shocks in the year of the survey and in the year prior to the survey	Height-for-age z-score, weight-for-height z-score, and weight-for-age z-score	- Exposure to more rainfall in the previous year was associated with increased weight-for-age z-score. - In contrast, excessive or above-average rainfall in the year of the survey was related to lower weight-for-age z-score.
Hong, 2025 [53]	25 million birth records between 2008 and 2021 in Mexico	Number of days of monthly exposure to different levels of wet-bulb temperature (> 24 °C)	Birth weight, birth length, and likelihood of birth weight < 2.5 kg during the in utero period	- Increased exposure to a wet-bulb temperature > 24 °C is associated with a reduction in birth weight, higher odds of low birth weight, and a reduction in birth length.
Islam et al., 2025 [54]	Children aged 0 to 23 months included in the National Nutrition Survey in Pakistan (*n* = 9,231 in 2011 and *n* = 20,656 in 2018)	Variation in average temperature and average rainfall	Length-for-age z-score and weight-for-length z-score	- Length-for-age and weight-for-age indexes were negatively associated with mean temperature and mean rainfall variation.
Kamiya, 2025 [55]	12,281 under-five-year-old children living in Mali, included in Mali Demographic and Health Surveys	Average monthly rainfall over periods of three months, six months, and one and two years before the survey; rainfall anomaly, monthly average temperature at three months, six months, and one and two years before the survey.	Stunting, underweight, and wasting according to weight-for-height	- The increase in rainfall was associated with a lower risk of wasting and underweight, but the extreme dry climate significantly increased the risk of wasting. - The protective effect of rainfall varied temporally between the South (3 months before the survey) and the North (up to 2 years before the survey) of Mali. - In the North region, the increase in average temperature increased the risk of stunting.
Khan et al., 2020 [56]	19,896 children from 0 to 59 months included in three rounds (2007, 2011 and 2014) of demographic and health surveys in Bangladesh.	Annual averages of rainfall and temperature.	Stunting	- The increase in average temperature was associated with lower odds of stunting. - Annual rainfall had a non-linear relationship with height-for-age: the likelihood of stunting increased with rainfall until it reached the peak at 4188 mm/year, and then decreased from this value. - In the model fit with all environmental variables, only rainfall remained significant, indicating overlapping effects.
LaPointe et al., 2024 [57]	3,016 live births in five rural districts of northern Ghana between 2015 and 2017.	Average weekly temperature (in Kelvin) in the 52 weeks prior to birth.	Low birth weight	- Weekly temperatures > 30 °C were associated with higher odds of low birth weight. The risk was higher in critical periods: weeks 2–14 and 35–44 before delivery. - In infants whose mothers received the LEAP 1000 benefit, the adverse effects of heat on birth weight were mitigated or absent.
Lazzaroni, Wagner, 2016 [58]	Children aged 12 to 60 months living in poor rural households participating in two rounds (*n* = 1,694 in 2009 and *n* = 2,116 in 2011) of a household panel survey conducted in eight regions of Senegal.	Perception of the household being affected by drought (in 2009 and 2011) and of the household being affected by cold waves (in 2009 and 2011)	Weight-for-age z-score	- The perception of drought occurrence was negatively associated with weight-for-age z-scores. - The perception of cold waves did not show a statistically significant association with weight-for-age.
López-Carr et al., 2015 [59]	Under-five-year-old children living in the households included in the Demographic and Health Survey of Eastern Africa’s Lake Victoria Basin (whose territory is administered by Tanzania, Kenya, and Uganda)	Variation in monthly rainfall from 1981 to 2012.	Stunting percentage	- There was a significant association between the increase in rainfall and stunting.
López-Carr et al., 2016 [60]	Under-five-year-old children living in households (*n* = 95,500) included in the Demographic and Health Survey (2012 − 2008) in countries in Eastern Africa’s Lake Victoria Basin (Burundi, Kenya, Rwanda, Tanzania, and Uganda).	Variation in rainfall from 1982 to 2012	Stunting percentage	- A positive association was found between the increase in rainfall and the percentage of stunted children. - Stunting is usually more prevalent in regions with the highest rate of rainfall over the years, especially in the northeast and southeast of Kenya.
MacVicar et al., 2017 [61]	3,197 newborns, included in the hospital records of the Bwindi Community Hospital, located in the Kanungu district, southwest Uganda, between 2012 and 2015	Mean daily temperature and mean number of days of precipitation >5 mm during the three trimesters of pregnancy and during gestation.	Birth weight	- Exposure to a higher rate of rainfall and higher mean temperature in the third trimester and during pregnancy was positively associated with birth weight.
Mank et al., 2021 [62]	1,439 children aged 7 to 60 months living in households in Burkina Faso, assessed in the Nouna Health and Demographic Surveillance Site, from 2017 to 2019	Rainfall variability score	Height-for-age z-score, weight-for-height z-score, stunting, and wasting according to the weight-for-height z-score	- Rainfall variability was associated with the reduction in the height-for-age z-score, indicating a higher risk of stunting. - Rainfall variability was positively associated with the weight-for-height z-score.
McMahon & Gray, 2021 [63]	222,572 children aged 24 to 59 months old included in 18 rounds of the Demographic and Health Survey, conducted between 1990 and 2018 in four countries in South Asia (Bangladesh, India, Nepal, and Pakistan)	Mean precipitation and mean temperature, standardized monthly temperature and rainfall anomalies, and counts of extreme days (hot, cold, dry, and rainy) during the prenatal period and the child’s first and second year of life.	Height-for-age z-score, severe stunting, and stunting	- Precipitation anomalies and a higher number of days with rainfall and drought extremes in the child’s first year of life were associated with a reduction in the height-for-age z-score and higher odds of stunting and severe stunting. - A greater number of days with rainfall extremes in the child’s second year of life was also associated with a reduction in height-for-age and higher odds of stunting. - More days of drought extremes during the prenatal period was associated with a reduction in height-for-age. - Precipitation anomalies in the child’s second year of life were associated with higher odds of severe stunting. - A greater number of cold days in the child’s first year of life was associated with lower odds of severe stunting. - Families without access to adequate sanitation and with low maternal education suffered more harmful effects.
Merwe et al., 2022 [64]	Under-five-year-old children included in two consecutive rounds of the Living Standards Measurement Study – Integrated Surveys on Agriculture (LSMS-ISA) conducted in Nigeria in 2010–2011, 2012–2013 and 2015–2016.	Variability in rainfall and temperature between post-planting and post-harvest stages	Stunting and underweight	- Increased temperature was associated with higher odds of stunting and underweight, especially in rural areas. - Increased rainfall was associated with lower odds of stunting, but it was not statistically associated with underweight.
Molina & Saldarriaga, 2017 [65]	86,021 newborns included in the Demographic and Health Surveys carried out between 1990 and 2013 in Bolivia, Colombia, and Peru	Temperature variability at 9, 6 to 8, 3 to 5 and 0 to 2 months before birth	Birth weight, occurrence of low birth weight, and perception of small size at birth	- The increase in temperature nine months before birth was associated with lower birth weight, higher odds of low birth weight, and increased perception of small size at birth. - Increased temperature at exposures from 6 to 8 months and from 3 to 5 months before birth was associated with lower birth weight.
Ngwira, 2020 [66]	5,149 children living in households included in the Malawi Demographic and Health Survey of 2015	Average annual temperature, annual rainfall	Stunting, wasting, and overweight according to the weight-for-height z-score.	- Increased rainfall was associated with higher odds of stunting and overweight according to the weight-for-height z-score. - Increased temperature was associated only with higher odds of wasting according to the weight-for-height z-score.
Nicholas et al., 2021 [67]	69,850 children aged 0 to 60 months living in the households included in the rounds of the Peruvian Demographic and Health Survey conducted in 1996, 2000, 2004–2008, 2009, 2011, and 2012	Temperature and rainfall anomalies during the prenatal period and at each year of life from 0 to 5	Height-for-age z-score	- Prenatal excess rainfall had opposite effects on the height-for-age z-score, associated with an increase at age 1–2 and a reduction at age 4–5. - Postnatal excess rainfall was significantly associated with increased height-for-age z-score at age 2–3. - The increase in postnatal temperature was not associated with height-for-age up age 3–4, but higher temperature levels were associated with increased height-for-age at age 4–5.
Niles, Brown, 2017 [68]	2095 households in smallholder farming communities that are typically low-income, included in household surveys of the Climate Change, Agriculture, and Food Security Program, collected between 2010 and 2012, in 14 locations in 12 countries in West and East Africa and in South Asia	Rainfall anomalies	Household food insecurity	- Rainfall variability is a critical factor in food insecurity, which increases as rainfall decreases (drought periods). - Households in ‘drier than average’ regions had the longest period of food insecurity (average of 3.81 months in the previous year). - Households in ‘wetter than average’ regions had the shortest period of food insecurity (average of 2.86 months in the previous year).
Omay et al., 2025 [69]	Data on the urban and rural population of Eastern African countries (Ethiopia, Kenya, and Uganda) of the IGAD (Intergovernmental Authority on Development) region	Standardized Precipitation Index	Food insecurity (cereal production, food prices, and undernourishment levels in the population of Ethiopia, Kenya, and Uganda)	- Rainfall has had a remarkable and inverse impact on undernourishment levels, indicating that precipitation contributes directly to food insecurity and vulnerability to drought.
Paul et al., 2012 [70]	Women of reproductive age (15–49 years) and children aged 6 to 59 months living in 331 households, with data collected between October and November 2008, from three coastal villages (Angulkata, Tatulbaria, and Charkashem), located in Bangladesh, which were severely affected by Cyclone Sidr	Cyclone event	Classification of the nutritional status of women of reproductive age considering their body mass index for age and classification of the nutritional status of children considering height-for-age, weight-for-age, and weight-for-height	- There were no statistically significant differences in the indicators of nutritional status of women of reproductive age and children when comparing the data of the three affected villages in the post-cyclone period with the data of the pre-cyclone national survey.
Petscavage et al., 2024 [71]	320,479 under-five-year-old children from 30 countries in Sub-Saharan Africa included in Health and Demographic Surveys (2010–2019)	Exposure to extreme weather events or natural disasters (droughts, floods, earthquakes, landslides, storms, and wildfires)	Stunting and wasting according to the weight-for-height z-score	- There was no statistically significant association between exposure to extreme weather events and increase in stunting. - However, exposure to natural disasters increased the risk of wasting.
Rahut et al., 2024 [72]	Children aged 0 to 59 months living in households located in rural areas included in the Demographic and Health Survey of six countries in South and Southeast Asia: India (2019-21), Bangladesh (2017–2018), Pakistan (2017–2018), Nepal (2016), Cambodia (2014), and Timor-Leste (2016).	Average annual rainfall and average land surface temperature.	Number of children with stunting, underweight, and wasting according to the weight-for-height z-score	- The relative temperature increase was positively associated with underweight, stunting, and wasting in India and Timor-Leste, but only with underweight in Nepal. - There was no association between temperature increase and nutritional outcomes in Bangladesh, Pakistan, and Cambodia. - The relative increase in rainfall was positively associated with underweight and wasting in Bangladesh; and with underweight and stunting in Timor-Leste. - There was no association between increased rainfall and nutritional outcomes in the other countries.
Randell et al., 2020 [73]	23,026 children aged 1 to 5 years living in the households included in the four rounds of the Demographic and Health Survey conducted in Ethiopia (2000, 2005, 2011 and 2016).	Average monthly rainfall during the prenatal period, early life (from birth to survey), during each trimester in utero, and each season in early life.	Height-for-age z-score, stunting, and severe stunting	- High temperatures in the first and third trimesters of pregnancy were associated with a higher risk of stunting and severe stunting. - Higher rainfall in the third trimester of pregnancy was associated with a higher risk of severe stunting. - Greater rainfall across a child’s life, and in the rainy seasons (kiremt and belg), was associated with higher height-for-age z-scores and lower risk of severe stunting. - Higher temperatures in early life, especially in the dry season (bega), were associated with a lower risk of stunting (potentially beneficial effect in mild climates). - There were significant nonlinear relationships between nutritional outcomes and climate variables.
Randell et al., 2021 [74]	11,029 households included in the 2016 Nepal Demographic and Health Survey	Monsoon rainfall anomalies in 2015	Food insecurity situation	- The effects of rainfall anomalies on food insecurity were modulated by the severity of the impact of the earthquake on the household. - Among households minimally impacted by the earthquake, low rainfall was associated with increased food insecurity. - In households with at least moderate tremors, the increase in rainfall was positively associated with increased food insecurity, especially in steep and mountainous areas.
Rodríguez-Llanes et al., 2011 [75]	Children aged 6 to 59 months from 191 flooded villages and 161 from non-flooded villages of Jagatsinghpur, Orissa State, India, assessed by a household survey conducted between October and November 2008	Flood exposure	Stunting, underweight, and wasting according to weight-for-height z-scores	- Children in flooded households were more likely to be underweight and stunted. - Children exposed to flooding during their first year of life were more vulnerable to stunting. - There was no difference in the prevalence of wasting (weight-for-height) among children exposed and not exposed to flooding.
Rodriguez-Llanes et al., 2016 [76]	879 children aged 6 to 59 months living in flooded and non-flooded communities in the Jagatsinghpur district, Odisha (India), one year after the major floods of 2008	Flood exposure	Stunting and severe stunting, wasting and severe wasting according to the z-scores, and wasting and severe wasting	- Flood exposure was associated with a higher prevalence of wasting. There was a higher prevalence of wasting among children exposed to flooding twice compared to children exposed once. The effect was stronger in children under one year.- There was a significant association with underweight, but the effect was less intense compared to wasting. There was no association with stunting.
Rojas Jr et al., 2023 [77]	Children between 24 and 59 months of age, living in households in Burkina Faso, assessed in four rounds of the Demographic and Health Survey (conducted in 1993, 1998–1999, 2003, and 2010).	Precipitation and temperature anomalies, standardized seasonal rainfall and temperature anomalies, and number of hot, cold, wet, and dry days during one year before birth, first and second year of life	Height-for-age z-score	- The effect of climate events on stunting is complex and non-linear, as confirmed by the greater explanatory power of models with interactions and quadratic terms. - The precipitation anomaly in the second year of life had a significant negative association with the height-for-age z-score. - Temperature in the first year had a positive and weak association with the height-for-age z-score. - There was a statistically significant effect of the interaction between precipitation and temperature anomalies.
Rosinger et al., 2023 [78]	118 household heads and 129 children (mean age = 6 years old) included in a longitudinal survey conducted with the Tsimane communities, in Bolivia, after the end of a flood (December 2013 to February 2014) and 8 weeks later	Flooding event	Food insecurity score; weight, body mass index, waist circumference, and sum of skinfolds among adults; weight-for-age z-score, height-for-age z-score, and body mass index-for-age z-score among children	- Two months after the flood, food insecurity remained high and without a significant change compared to the period just after the end of the flood. - Adults showed significant reductions in measures of adiposity. - Children showed increased weight-for-age z-scores and body mass index-for-age z-score, indicating buffering from food stress.
Sam et al., 2019 [79]	157 farming households located in drought-prone areas in the District of Balangir, in the state of Odisha, eastern India.	Perception of drought factors between 2009 and 2014: perception of the impact of climate variability on agriculture; drought-induced stress; drought-induced unemployment; drought-induced disease among family members; and drought-induced deterioration of the income-generating local environment.	Food Security Index	- The perception of the impact of climate variability on agriculture, drought-induced stress, and drought-induced unemployment had negative effects on household food security. - Stress was found to be the factor with the greatest negative impact on food security. -The factors drought-induced unemployment, drought-induced diseases, and deterioration of the income-generating environment did not show significant association with food security.
Sam et al., 2021 [80]	220 households in the four villages (Beguniabasta, Gopalpur, Alanda, and Manijanga) of the Puri district, Odisha (rural India)	Perceived flood impacts: losses of property due to floods, presence of flooding stress, average rainfall variation between 2009 and 2014, and income affected by environmental deterioration due to flooding.	Food Security Index	- The perception of property losses due to flooding was inversely associated with the food security index, indicating that losses aggravated food insecurity. - No statistically significant associations were found between the food security index and: presence of flooding stress, mean rainfall variability (2010–2015), or income affected by environmental deterioration.
Sandler, Sun, 2024 [81]	Children aged 0 to 59 months living in households included in the Demographic and Health Surveys of Kenya and Nigeria (2003, 2008–2009, 2013, 2014) (*n* = 48,086 Nigerian children and 28,421 Kenyan children)	Average total monthly rainfall during the growing season prior to the child’s birth; rainfall anomaly, average monthly maximum temperature during the growing season prior to the child’s birth; temperature anomaly	Prevalence of stunting and prevalence of wasting according to the weight-for-height z-score	- In Kenya, the increase in rainfall in the previous growing season was associated with a lower prevalence of wasting and a higher prevalence of stunting. The increase in temperature in the growing season was associated with increased wasting in the following year and an inverse relationship to stunting. - In Nigeria, the increase in temperature during the growing season was associated with increased wasting in the following year. Temperature anomaly is associated with a lower prevalence of wasting.
Scharadin et al., 2023 [82]	All U.S. Counties, based on datasets from the Farm Economy Branch of the U.S. Department of Agriculture Economic Research Service for the years 2010/2011 and 2015/2016.	Count of the following extreme weather events between 2013 and 2017: hurricane/tropical storm, wildfire, tornado, winter weather, severe storm/thunder, lightning, coastal flooding, flooding, heat, and wind	Density of grocery stores, supermarkets, and convenience stores, and proportion of low-income population, with low access to grocery stores/supermarkets, and no access to a car	- Counties with a higher number of weather events showed lower density of grocery stores, convenience stores, and supermarkets, and a lower proportion of the vulnerable population had food accessibility. - The effects were more intense in counties with better levels of initial accessibility (upper quartiles), and localized events had a stronger association with the reduction of food availability than large-scale events.
Silva et al., 2025 [83]	92 municipalities in Rio Grande do Sul that had at least 1 food establishment affected by the floods of May 2024	Flood intensity and extent, flooded area versus non-flooded area	Proportion of establishments affected (categorized by type and group of establishments)	- The floods affected 17.5% of food establishments in the sample, concentrating on nine municipalities that lost more than 45% of their businesses. The establishments selling fresh and minimally processed foods were the most affected (especially fishmongers), compared to establishments selling ultra-processed foods.
Shaw et al., 2020 [84]	Data from 259,627 under-five-year-old children from 640 districts in India, included in the National Family Health Survey 2015–2016.	Scaled drought condition index	Stunting, wasting according to the weight-for-height z-score, and underweight	- The agricultural drought index is negatively associated with stunting, which means that districts with higher drought levels have a higher prevalence of this outcome. - No statistically significant associations were found between the index and wasting and underweight outcomes.
Shively, 2016 [85]	11,946 under-five-year-old children living in households included in the Demographic and Health Survey of 2006 and 2011 conducted in Nepal (*n* = 7,572) and Uganda (*n* = 4,374)	Rainfall and temperature anomalies during the gestational period, first year of life, and survey year	Length-for-age z-score and weight-for-length z-score	- An increase of 1 standard deviation in growing season rainfall was associated with an increase of about 4 to 13% in height-for-age and 1 to 7% in weight-for-height z-scores, respectively. - The effect of rainfall was greater in Nepal than in Uganda (owing to lower average rainfall and higher amplitude in Nepal). - In Nepal, temperature was negatively associated with the weight-for-height z-score.
Skoufias & Vinha, 2012 [86]	Children between 12 and 47 months living in rural areas of 169 municipalities in Mexico included in the National Nutrition Survey, conducted at the end of 1999	Negative and positive rainfall and temperature anomalies	Height-for-age z-score	- A positive rainfall anomaly was associated with a reduction in the height-for-age z-score, regardless of region or altitude. - Negative temperature anomalies had a negative impact on height-for-age z-scores in central and southern regions and at higher altitudes. - Positive temperature anomalies had no significant impact on average, but they negatively affected vulnerable sub-populations: boys, children between 12 and 23 months, and children of less educated mothers.
Sultan et al., 2025 [87]	Data on children aged 6 to 59 months living in Niger, collected from the Standardized Monitoring and Assessment of Relief and Transition surveys (2005–2022), Demographic and Health Surveys, and Multiple Cluster Indicator Surveys (1992–2000)	Rainfall indexes (cumulative rainfall from June to September; onset of rainfall; cessation of rainfall; duration of the rainy season; number of dry spells) and temperature indexes (mean surface temperature; minimum temperature; maximum temperature; percentage of days with maximum temperature > 90º percentile; percentage of days with minimum temperature > 90º percentile; heat wave duration index).	Prevalence of Global Acute Malnutrition and severe acute malnutrition, considering the weight-for-height z-score	- Regions with lower summer rainfall in the previous year tend to have higher rates of malnutrition (Global Acute Malnutrition indicator). This relationship does not exist when considering rainfall and malnutrition in the same year, but it becomes more pronounced when using crop yield simulations of the previous year. - The correlations between malnutrition and rainfall are stronger on a regional scale than on a national scale. - The Agadez region has low rates of malnutrition despite the occurrence of drought, owing to favorable socioeconomic factors and diversified livelihoods, which mitigate climate risks. - The other climate variables did not show a significant association with the malnutrition indicators.
Thiede, Gray, 2020 [88]	4,812 children aged 0 to 23 months and 7,459 children aged 24 to 59 months in 304 Indonesian communities participating in the Indonesian Family Life Survey, in 13 provinces.	Average delays in monsoon onset, daily records of rainfall (mm/day) and temperature (ºC) during the prenatal period, 0 to 11 months of life, and 12 to 23 months of life (for height-for-age), and the last 12 months prior to the survey (for weight-for- height).	Height-for-age z-score in children aged 24 to 59 months and weight-for-height z-score in children aged 0 to 23 months.	- Delays in monsoon onset during pregnancy caused a significant decline in height-for-age z-scores and weight-for-height z-scores. - Negative effects on height-for-age were stronger in boys, farming families, urban residents, and people living outside Java Island. - Negative effects on weight-for- height were stronger in girls, non-farming families, rural residents, and residents on Java Island. - Average temperature and average rainfall rates did not have significant effects on the indicators for children.
Thiede, Strube, 2020 [89]	182,272 records of under-five-year-old children living in households included in 39 rounds of the Demographic and Health Survey conducted in 18 countries in Sub-Saharan Africa between 1990 and 2015.	Temperature and precipitation anomalies in the previous 12 months	Weight-for-height z-score and wasting according to the weight-for-height z-score	- Temperature anomaly is positively associated with lower weight-for-height z-score and higher odds of wasting. - An equivalent decline of two standard deviations in precipitation is associated with a reduction in weight-for-height z-scores.
Tiwari et al., 2017 [90]	13,683 children aged 0 to 59 months living in rural Nepal, included in the Demographic and Health Survey conducted in 2001, 2006, and 2011.	Monsoon rainfall shocks in the current season, in the previous season, and in the child’s first years of life	Weight-for-height z-score and height-for-age z-score	- Increased rainfall in previous monsoon seasons was associated with an increase in weight-for-height z-scores. - The increase in rainfall in the current season was associated with the reduction of the child’s weight-for-height z-score. - Children who experienced heavy rains in their second year of life are taller on average, but this effect was not observed when they were 5 years old.
Tusting et al., 2020 [91]	656,107 children aged 0 to 5 years living in households included in Demographic and Health Surveys and Malaria Indicator Surveys conducted between 2003 and 2016 in 29 countries in Sub-Saharan Africa.	Monthly mean daytime land surface temperature	Stunting, wasting according to the weight-for-height z-score, concurrent stunting with wasting, and underweight	- Compared with temperatures < 30 °C, mean daytime temperatures > 35 °C were associated with lower odds of stunting (greater effect in children aged 2 to 5 years). - The odds of wasting were higher where the mean daytime temperature exceeded 35 °C, with similar results in all age groups. - The odds of underweight were higher where the mean daytime temperature exceeded 35 °C (slightly higher effect in children < 2 years). - The odds of concomitant underweight with wasting were higher where the mean daytime temperature exceeded 35 °C (greater effect on children aged 2 to 5 years).
Xiong et al., 2008 [92]	301 newborns under care at prenatal clinics in the United States, between January 2006 and June 2007.	High exposure to hurricanes≥ ( 3 events)	Low birth weight	- High exposure to hurricanes was associated with a more than three-fold risk of babies with low birth weight. - There was a tendency for increased low birth weight rates due to an increasing number of severe hurricane events or experiences.
Yeboah et al., 2022 [93]	12,919 under-five-year-old children included in a survey with 6,890 households in 526 villages in 24 rural districts of Burkina Faso.	Average rainfall exposure (mm) from conception to the survey month	Height-for-age z-score.	- Average lifetime rainfall exposure had a negative and significant association with height-for-age z-scores. - The ratio is non-linear: rainfall (up to approximately 75 mm) decreased height-for-age, and after such point, the relationship reverses - The negative association was significant only in children aged 0 to 23 months and for both boys and girls. - The strongest effect of the negative association was found in the second poorest wealth quintile.
Yuan et al., 2025 [94]	23,182 singleton pregnant women participating in a cohort study in Beijing, China	Apparent temperature (air temperature combined with relative humidity), baseline temperature, maximum and minimum temperature, from the first day of the last menstrual period to each ultrasound examination.	Parameters for fetal growth in utero: fetal head circumference, abdominal circumference, femur length, and estimated fetal weight	- Both high and low temperatures had negative effects on fetal growth in all parameters. The negative effects were more intense during the early stages of pregnancy (at ≤ 23 weeks). - While fetal head circumference and body measurements exhibited the classic U-shape response, estimated fetal weight and femur length showed greater sensitivity to high temperatures.

Source: Authors

### Methodological Aspects of the Reviewed Studies

In general, the studies used data from population surveys or other secondary data sources (such as hospital databases, etc.) to investigate the anthropometric outcomes of individuals and food security in households. Based on georeferenced data of households or household clusters (in the case of population surveys), data on the outcomes of interest were linked to data on climate events. Table S2 of the Online Resource 1 shows the main methodological aspects of the reviewed studies.

For climate events, almost all studies (*n* = 73, 93.6%) used objective measures from secondary data from official databases to collect information on climate events while only five studies evaluated subjective measures (6.4%). Two of such studies using subjective measures, evaluated householder perceptions of the occurrence of climate events, such as droughts, erratic rainfall, and cold waves [45, 58], and three studies evaluated respondents’ perception of the consequences of climate events on the household (for example, property losses, stress, income losses, etc.) [19, 79, 80] (Table 3).

The studies that addressed extreme weather events usually used a comparative methodology, both between exposure and non-exposure to events, and through analysis before and after the occurrence of events (Table 3 and Table S2 of the Online Resource 1).

The studies that evaluated climate variability focused on the evaluation of temperature and precipitation anomalies to estimate the associations of their extremes on anthropometric outcomes and food security. For temperature and precipitation anomalies, the studies often used standardized deviations obtained through the difference between the current measurement and the average of a previous longer period (Table 3 and Table S2 of the Online Resource 1).

Most studies that evaluated precipitation and temperature variability, sought to analyze prior exposure to these climate events, using data from specific periods, such as conception, gestation (in utero period), first months/years of life, and the 12-month period prior to the survey, to evaluate their associations with outcomes that were observed later (Table 3 and Table S2).

### Main Findings

Standardized indicators of anthropometric outcomes (continuous variables) and undernutrition (acute or chronic) showed mixed results depending on the outcome and the evaluated climate exposure.

Most studies found that precipitation anomalies or extremes were associated with a reduction of height-for-age [18, 32, 35, 42, 43, 62, 63, 77, 86], while the results for this climate exposure are mixed for the other anthropometric outcomes (Table 3).

It was found that the increase in mean temperature and the occurrence of high temperature extremes were associated with a reduction in weight-for-height [28, 30, 31, 66, 72, 73, 81, 85, 88, 89, 91], weight-for-age z-score [28, 31, 54, 66, 72, 73, 91] and birth weight [29, 33, 39, 47, 48, 53, 57] in most studies, while mixed results were reported for height-for-age [27, 67, 73] (Table 3).

There were few studies that evaluated the association between extreme weather events and standardized anthropometric indexes and undernutrition indicators, and mixed results were found.

Only 6 studies evaluated anthropometric measurements of adiposity [24, 25, 37, 66, 70, 78], and diverging findings were reported. One study found that exposure to a higher number of days with temperatures above 30 °C was associated with a small increase in body mass index and higher odds of obesity in adults [25]. Among children, one study found that infants exposed to the highest temperature quintile presented a higher risk of overweight at one year of age and at two years of age, compared to those exposed to the lowest temperature quintile [24], and another study showed that an increase in rainfall was associated with a higher probability of overweight according to weight-for-height [66].

For extreme weather events, one study found that the occurrence of flooding was associated with a reduction of adiposity measures in adults (such as weight, BMI, WC, and skinfolds), while there was an increase in BMI-for-age z-scores in children [78]. However, other studies found no association between the occurrence of cyclones in children and adults [70] and of flooding in children [37] and adiposity measures.

Few studies evaluated the outcome of food security, and the majority evaluated the association with rainfall variability, finding mixed results [3, 19, 36, 41, 68, 69, 74]. A small number of studies evaluated the relationship of extreme weather events such as drought and flooding with food insecurity, and most of them found a positive association with food insecurity [19, 78–80] (Table 3).

Only three studies evaluated the food environment as an outcome, and they focused primarily on the physical and logistical disruptions linked to climate events. Scharadin et al. [82] found that areas with a higher number of extreme weather events showed a lower density of food retail environments and lower food access and availability, and localized events (tornadoes, floods) showed a stronger association with a reduction of access and availability than broad events (hurricanes). Silva et al. [83] found that floods affect a significant portion of food establishments, and establishments that sell fresh and minimally processed foods are the most vulnerable and affected. Although most establishments that were affected sell ultra-processed food, the destruction of the infrastructure for selling healthy food significantly reduced physical accessibility to food in flooded areas [83]. In addition, the findings highlighted the vulnerabilities in food access logistics, showing that disruptions in road networks hinder access to food pantries by vulnerable families [34] (Table 3).

**Fig. 3:**
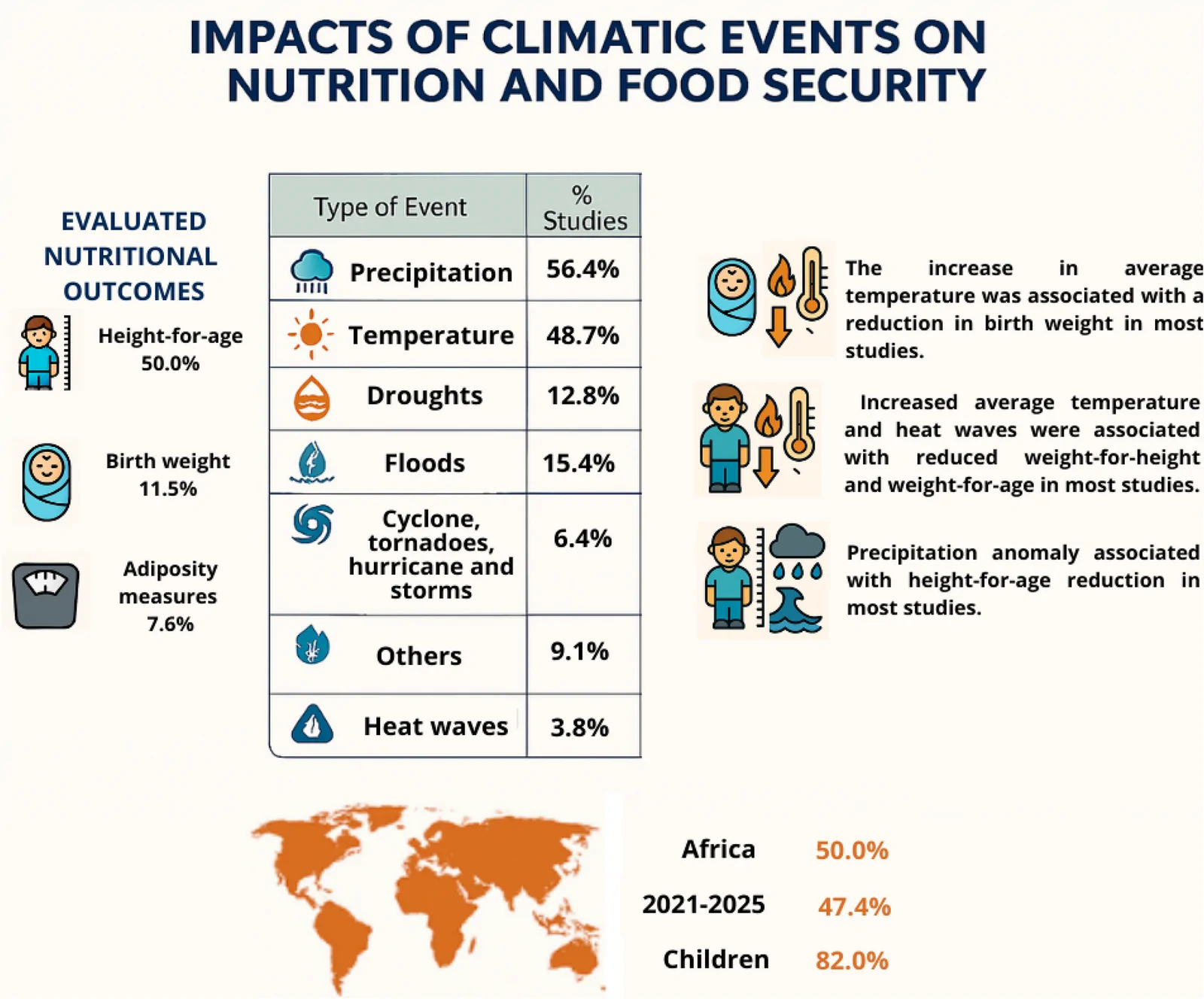
Summary of geographical distribution, primary exposures, and nutritional outcomes of the included studies

Figure 3 is a visual summary of the main findings of this review, highlighting the most prevalent anthropometric outcomes, the types of climate events, and their respective associations.

## Discussion

This scoping review mapped scientific evidence on the associations of extreme weather events and climate variability with anthropometric outcomes, food security, and food environments. The reviewed studies focused mostly on outcomes related to child undernutrition, especially among under-five-year-old children. This finding underscores that this age group is biologically vulnerable to the consequences of climate events, and is prioritized in traditional systems of nutrition surveillance [35, 63, 73, 95]. However, the lack of studies with adolescents, the elderly, and pregnant women represents an important research gap.

Most studies analyzed associations of temperature and precipitation variability, as well as extreme weather events, with undernutrition, but the strength and direction of the relationships vary depending on the outcome assessed.

The association of precipitation anomalies with stunting points to a complex relationship that transcends the mere occurrence of the event, requiring the analysis of the timing of exposure and its cumulative nature. Studies show that the relationship with growth indicators depends on the timing of exposure. This link is mainly mediated by agricultural production and long-term food security [35]. Favorable rainfall in previous years, for example, can act as a protective factor in rural areas, because it guarantees higher food stocks, improves agricultural yields, and allows greater food diversity and lower food prices [52, 55].In contrast, drought or excessive rainfall at harvest disrupts the availability and immediate access to food, which can be aggravated by structural factors such as low maternal education, lack of basic sanitation, and poor housing [78, 79]. In addition, excess rain may favor the occurrence of diarrheal diseases, which may compromise nutritional status. This nuance suggests that adaptation and mitigation strategies should consider cumulative and temporal exposure rather than just a single climate shock. In addition to this complexity, there are nonlinear effects, in which the greatest nutritional losses are associated with extreme conditions, rather than with moderate variations in the climate [44, 45, 73, 77, 84].

Temperature rise, in turn, showed a greater association with acute undernutrition (wasting and underweight) [24, 28, 66, 72, 81, 85, 91]. This pattern of acute impact extends to the gestational period, where temperature was consistently associated with low birth weight [29, 33, 39, 46, 48, 53, 57, 65, 94]. Studies suggest that heat stress alters maternal thermoregulation, inducing increased energy expenditure and dehydration. In turn, this can impair placental function, and reduce blood flow and transport of nutrients and oxygen to the fetus, resulting in fetal growth restriction [48, 53, 94]. This association is critical, since it highlights the vulnerability of children’s nutritional status from conception onward, suggesting that gestational heat stress may be linked to intergenerational impacts with long-term metabolic and cognitive consequences [26, 48]. In addition, high temperature extremes can favor vector proliferation, increasing the incidence of infectious and diarrheal diseases, thus negatively affecting nutritional status [54, 55, 73, 86].

Despite the recognized centrality of food security for climate resilience, only 12.8% (*n* = 10) of the reviewed studies evaluated this outcome, and they found mixed results. Some studies showed that flooding and precipitation extremes were associated with worse food security, especially among populations dependent on subsistence agriculture [3, 36, 41, 68, 87], while other studies did not find a statistically significant association. This divergence across studies largely stems from differences in exposure metrics, study designs, and local environmental contexts. Studies assessing primary, self-reported household perceptions of climate shocks—such as property loss, agricultural disruption, and psychological stress following floods or droughts—consistently demonstrated severe adverse impacts on household food security [19, 78–80].

Conversely, studies utilizing secondary meteorological indices (such as precipitation anomalies or drought scores) yielded contrasting findings, as the direction of association depended heavily on baseline agro-ecological conditions [36, 68]. For instance, increased rainfall improved food security in arid zones by boosting crop yields, whereas excessive rain exacerbated food insecurity in mountainous or disaster-prone settings [36, 74]. Furthermore, compound shocks and varying levels of local infrastructure strongly moderate how climate disruptions translate into household food access [69, 74].

Only three studies directly analyzed the food environment as an outcome, despite the increasing importance of this environment in determining dietary patterns. The retail food environment encompasses built environments, where consumers access food through formal and informal markets, as well as factors such as physical accessibility, prices, and food availability [17]. Because local food retail environments are deeply integrated into broader food systems, climate events ripple across supply chains—disrupting agricultural production, transportation, and market infrastructure.

In the reviewed studies conducted in the Americas, extreme weather events were linked to severe logistic and physical damage to food retail. These systemic supply chain disruptions impair normal market operations, drive up prices, and acutely restrict physical access to fresh, minimally processed foods. Crucially, when local food environments degrade, they often catalyze dietary shifts toward cheap, shelf-stable, energy-dense ultra-processed products. This pathway illustrates how climate-driven food system disruptions directly compromise local food environments, worsening dietary quality and exacerbating the double burden of malnutrition in socioeconomically vulnerable populations [12, 82, 83, 96].

In addition, the indirect consequences of disasters, such as loss of housing, and closure of schools and primary health care units, disrupts health care provision, supply of school meals, and access to social welfare. In temporary shelters or forced displacement contexts, children are even more susceptible to undernutrition and infectious diseases [31, 97].

A relevant finding of this review is the scarcity of studies on obesity and the double burden of malnutrition in climate change contexts. Only 7.6% of the studies evaluated adiposity outcomes, reflecting a major focus on malnutrition. However, this pattern results from the global research and funding agenda, which historically prioritizes low- and middle-income countries in Africa and Asia. This concentration is due to the persistence of undernutrition, especially among under-five-year-old children. Africa is one of the most vulnerable continents, with extreme weather events affecting millions of people and resulting in significant loss of life and livelihoods [98]. Similarly, Asian countries with dense populations that are highly vulnerable to climate events, such as heat waves and floods, are also recognized as a focal point for health and climate research [99].

It is noteworthy that the climate crisis is a catalyst for the double burden of malnutrition, because food insecurity linked to climate events exacerbates both undernutrition and overweight, which reinforces the need to adopt approaches that articulate food, environment, and health from the perspective of the global syndemic [100].

In this scenario, the scarcity of studies conducted in Latin America is a critical gap. While the research agenda historically prioritizes Africa and Asia, where climate vulnerability is extreme, there have been research initiatives in other locations. Recent global movements, such as the Lancet Countdown, have driven the expansion of health and climate research into Latin America, which can also explain the increase in scientific production over the past five years [96, 99]. This agenda is urgent, in view of the advancement of nutrition transition and the increase in climate vulnerability in the region, which demands studies centered around Latin America’s social and environmental context.

In the present review, there was a smaller number of studies that included extreme weather events. In this context, considering the increase in frequency and intensity of extreme weather events in recent years [101], the research agenda needs to look further into them to better evaluate their immediate and cumulative relationships with anthropometric outcomes, food security, and the food environment.

The findings should be interpreted in the light of the methodological limitations of this review. Although the search was carried out without restrictions of date or geographical location of the studies, there was a limitation regarding language: only publications available in English, Spanish or Portuguese were included. This choice may have excluded relevant studies published in local languages, especially in regions such as Asia and Africa; thus, it is a possible source of bias in the synthesis of results. In addition, the exclusion of gray literature may result in the absence of relevant publications, especially in contexts of policies and interventions.

Despite these limitations, this study makes an important advance in mapping studies on the association of climate events with anthropometric outcomes, food security, and the food environment. From a methodological perspective, the body of evidence presents varying levels of strength. A notable strength is the inclusion of robust longitudinal panels and prospective cohorts [22, 45, 92], as well as quasi-experimental designs. However, while the predominant study design relies on cross-sectional health surveys, it is crucial to highlight that most of these studies evaluate previous climate exposure by utilizing historical, secondary climate data. For climate variability, studies typically assess exposure over the preceding months or years to calculate specific climatic indices, such as precipitation and temperature anomalies across different time windows [24, 42, 85]. Similarly, for extreme weather events, researchers frequently compare scenarios before and after the event, or contrast affected versus unaffected locations [18, 21, 75]. This methodological approach strengthens the temporal inference of these associations, despite the cross-sectional nature of the anthropometric and health data.

Furthermore, it is important to acknowledge the inherent methodological limitations and potential sources of bias within this observational literature. First, several studies rely on aggregate regional data (ecological designs), introducing the risk of ecological fallacy when inferring individual-level nutritional outcomes. Second, exposure misclassification is a frequent concern across cross-sectional studies, as macro-level meteorological metrics, such as satellite rainfall data, may not accurately capture the precise micro-environment or the actual lived exposure of the populations [62, 87]. Finally, given the complex social determinants of nutrition, residual confounding remains a significant challenge, as many primary studies could not fully adjust for all intersecting socioeconomic vulnerabilities. The systematization of this emerging field provides an important basis for designing more specific research.

These findings suggest an urgency to advance the production of evidence, focusing on the identified gaps. It is essential to conduct more studies on extreme weather events, adiposity-related outcomes, and their impacts on food security and the food environment. Future studies should expand research into other life cycles and their methods should go beyond the assessment of pre- and post-exposure impacts. Furthermore, it is essential to consider the intersectionality of social factors and the life cycles of vulnerable populations, ensuring that adaptive responses are evaluated in different contexts.

Owing to more intense climate events and their overlap with food insecurity and health inequalities, there is an urgent need for responses aligned with the concept of global syndemic [12]. The findings reinforce the need to strengthen cross-sector public policies that address healthy, resilient, and sustainable food systems, foster health promotion and advance social justice. Crucially, evidence in this domain must be translated into triple-duty actions that directly align climate adaptation with resilient food systems. In terms of public policy, this entails strengthening nutritional surveillance within primary health care to identify and respond early to nutritional risks in climate-vulnerable territories.

Furthermore, strengthening climate-resilient social protection — such as expanding school feeding programs anchored in local agricultural supply chains — serves as a crucial buffer against supply disruptions. Simultaneously, regulatory interventions in local food environments are needed to cushion against food price volatility, protecting access to nutrient-dense foods while mitigating the market expansion of ultra-processed products during environmental crises. Ultimately, integrating health, social welfare, agriculture, environment, and education is essential to foster food and nutrition resilience, ensuring the human right to adequate food and addressing structural inequalities.

## Conclusion

The findings of this scoping review show significant associations of climate variability with anthropometric outcomes of vulnerable populations, especially children under five years of age. The focus of the current literature is on child undernutrition; chronic undernutrition is associated with precipitation anomalies and acute undernutrition is associated with higher average temperature. The findings highlight gaps in studies that have evaluated extreme weather events, outcomes related to adiposity, food security, and the food environment, and studies that have included other life cycles, reinforcing the urgent need for integrated approaches within the framework of the global syndemic.

## Key References

- Gomes SM et al. (2024). Nexus among climate change, food systems, and human health: an interdisciplinary research framework in the Global South. Environmental Science & Policy, 161, 103,885. https://doi.org/10.1016/j.envsci.2024.103885.
  - ○ This paper provides an interdisciplinary research framework tailored to the Global South that operationalizes and expands the premises of the Global Syndemic. By explicitly encompassing the food environment, the study highlights the urgency of addressing systemic vulnerabilities at the intersection of climate change, food systems, and human health.
- Fanzo J et al. (2025). Climate Change, Extreme Weather Events, Food Security, and Nutrition: Evolving Relationships and Critical Challenges. Annual Review of Nutrition, 45, 335–360. https://doi.org/10.1146/annurev-nutr-111324-111252.
  - ○ This comprehensive review updates the pathways of the Global Syndemic by proposing an interconnected systems approach that integrates diet quality, compound events, and the bidirectional relationship between climate change and obesity. The study details how these exposures influence multiple outcomes, highlighting the urgent need for multisectoral solutions.

## Supplementary Information

Below is the link to the electronic supplementary material.

Supplementary Material 1 (DOCX 78.2 KB)

## Data Availability

No datasets were generated or analysed during the current study.

## References

[1] Gholami R, Watson R, Hasan H, Molla A, Bjorn-Andersen N. Information systems solutions for environmental sustainability: how can we do more? J Association Inf Syst. 2016;17(8):52–36. 10.17705/1jais.00435.

[2] Ebi KL, Helmer M, Vainio J. The health impacts of climate change: getting started on a new theme. Prehosp Disaster Med. 2008;23:s60–4. 10.1017/S1049023X00021269. 18935961

[3] Drysdale RE, Moshabela M, Bob U. A creeping phenomenon’: the association between rainfall and household food insecurity in the District of iLembe, KwaZulu-Natal. Climate Dev. 2021;13:128–38. 10.1080/17565529.2020.1737795.

[4] Thornton PK, Ericksen PJ, Herrero M, Challinor AJ. Climate variability and vulnerability to climate change: a review. Glob Change Biol. 2014;20:3313–28. 10.1111/gcb.12581. PMC4258067 24668802

[5] Romanello M, Walawender M, Hsu SC, Moskeland A, Palmeiro-Silva Y, Scamman D, Ali Z, Ameli N, Angelova D, Ayeb-Karlsson S, et al The 2024 report of the lancet countdown on health and climate change: facing record-breaking threats from delayed action. Lancet. 2024;404:1847–96. 10.1016/S0140-6736(24)01822-1. PMC7616816 39488222

[6] Fanzo J, et al. Climate change, extreme weather events, food security, and nutrition: evolving relationships and critical challenges. Annu Rev Nutr. 2025;45:335–60. 10.1146/annurev-nutr-111324-111252. 40239306

[7] FAO. (2020). The state of food security and nutrition in the world 2020. Food and agriculture organization of the United Nations. Knowledge Repository. https://openknowledge.fao.org/bitstreams/726c7c55-89a4-426e-958c-d5f19bd12a21/download

[8] IPCC (Intergovernmental Panel on Climate Change). Managing the risks of extreme events and disasters to advance climate change adaptation. a special report of working groups I and II of the intergovernmental panel on climate change. Cambridge, UK, and New York, NY, USA: Cambridge University Press; 2012.

[9] Abbass K, Qasim MZ, Song H, Murshed M, Mahmood H, Younis I. A review of the global climate change impacts, adaptation, and sustainable mitigation measures. Environ Sci Pollut Res. 2022;29:42539–59. 10.1007/s11356-022-19718-6. PMC8978769 35378646

[10] Mulondo M, Hege A, Tsoka-Gwegweni J, Ndirangu J. Effect of climate change on the health and nutritional status of children and their families in Africa: scoping review. PLOS Glob Public Health. 2025;5:e0004897. 10.1371/journal.pgph.0004897. PMC12258551 40658712

[11] FAO, IFAD, UNICEF, WFP, WHO., (2025). The State of Food Security and Nutrition in the World 2025. FAO. 10.4060/cd6008en

[12] Swinburn BA, Kraak VI, Allender S, Atkins VJ, Baker PI, Bogard JR, Brinsden H, Calvillo A, De Schutter O, Devarajan R, et al. The global syndemic of obesity, undernutrition, and climate change: The Lancet Commission Report. Lancet. 2019;393:791–846. 10.1016/S0140-6736(18)32822-8. 30700377

[13] United Nations Brazil. Sustainable Development Goals. https://brasil.un.org/pt-br/sdgs.

[14] Tricco AC, Lillie E, Zarin W, O’Brien KK, Colquhoun H, Levac D, Moher D, Peters MDJ, Horsley T, Weeks L, et al. PRISMA extension for scoping reviews (PRISMA-ScR): checklist and explanation. Ann Intern Med. 2018;169:467–73. 10.7326/M18-0850. 30178033

[15] World Health Organization. WHO Child Growth Standards: Length/height-for-age, weight-for-age, weight-for-length, weight-for-height and body mass index-for-age: methods and development. Geneva: World Health Organization; 2006.

[16] FAO. Rome Declaration on World Food Security and World Food Summit Plan of Action. Rome: Food and Agriculture Organization of the United Nations; 1996.

[17] Downs SM, Ahmed S, Fanzo J, Herforth A. Food environment typology: advancing an expanded definition, framework, and methodological approach for improved characterization of wild, cultivated, and built food environments toward sustainable diets. Foods. 2020;9:532. 10.3390/foods9040532. PMC7230632 32331424

[18] Aguilar A, Vicarelli M. El Niño and children: medium-term effects of early-life weather shocks on cognitive and health outcomes. World Dev. 2022;150:105690. 10.1016/j.worlddev.2021.105690.

[19] Ahmad D, Shah SZA, Afzal M. Flood Hazards Vulnerability and Risk of Food Security in Bait Community Flood-Prone Areas of Punjab Pakistan. SDGs Achievement Threat Environ Sci Pollut Res. 2022;29:88663–80. 10.1007/s11356-022-21683-z. 35836043

[20] Ahmed K, Agho K, Page A, Arora A, Ogbo F. On behalf of the Global Maternal and Child Health Research Collaboration (GloMACH). Mapping Geographical Differences and Examining the Determinants of Childhood Stunting in Ethiopia: A Bayesian Geostatistical Analysis. Nutrients. 2021;13:2104. 10.3390/nu13062104. PMC8234472 34205375

[21] Ahsanuzzaman IMQ. Children’s Vulnerability to Natural Disasters: Evidence from Natural Experiments in Bangladesh. World Dev Perspect. 2020;19:100228. 10.1016/j.wdp.2020.100228.

[22] Alterman N, Nevo D, Calderon-Margalit R, Youssim I, Weinstein B, Kloog I, Hauzer M, Raz R. Ambient Temperature Exposure and Rapid Infant Weight Gain. Int J Epidemiol. 2024;53:dyae134. 10.1093/ije/dyae134. 39403064

[23] Alterman N, Nevo D, Calderon-Margalit R, Youssim I, Kloog I, Hauzer M, Raz R. Ambient Temperature and Indicators of Overweight during Infancy and Early Childhood: A Population-Based Historical Cohort Study. Environ Res. 2025;282:121983. 10.1016/j.envres.2025.121983. 40447025

[24] Amegbor PM, Lapurga K, Carr E, Guerrero M, Babayode O, Crisci SE, Fang XY, Siddeky A, Quansah R. Exploring the Effect of Early-Life Climate Anomalies on Child Growth in Sub-Saharan African Context: Insight from the Demographic & Health Survey. Sci Total Environ. 2025;983:179658. 10.1016/j.scitotenv.2025.179658. 40398169

[25] Ampofo A, Churchill REA, Awaworyi Churchill S. When the Thermometer Rises, the Scales Tip: Extreme Temperature Exposure and Obesity. Econ Hum Biology. 2025;58:101516. 10.1016/j.ehb.2025.101516. 40749328

[26] Baez JE, Kshirsagar V, Skoufias E. Drought-Sensitive Targeting and Child Growth Faltering in Southern Africa. World Dev. 2024;182:106702. 10.1016/j.worlddev.2024.106702.

[27] Baffour B, Aheto JMK, Das S, Godwin P, Richardson A. Geostatistical modelling of child undernutrition in developing countries using remote-sensed data: evidence from Bangladesh and Ghana demographic and health surveys. Sci Rep. 2023;13:21573. 10.1038/s41598-023-48980-y. PMC10703913 38062092

[28] Baker RE, Anttila-Hughes J. Characterizing the contribution of high temperatures to child undernourishment in Sub-Saharan Africa. Sci Rep. 2020;10:18796. 10.1038/s41598-020-74942-9. PMC7606522 33139856

[29] Basu R, Rau R, Pearson D, Malig B. Temperature and term low birth weight in California. Am J Epidemiol. 2018;187:2306–14. 10.1093/aje/kwy116. 29901701

[30] Blom S, Ortiz-Bobea A, Hoddinott J. Heat exposure and child nutrition: evidence from West Africa. J Environ Econ Manag. 2022;115:102698. 10.1016/j.jeem.2022.102698.

[31] Bonell A, Vicedo-Cabrera AM, Moirano G, Sonko B, Jeffries D, Moore SE, Haines A, Prentice AM, Murray KA. Effect of heat stress in the first 1000 days of life on fetal and infant growth: a secondary analysis of the ENID randomised controlled trial. Lancet Planet Health. 2024;8:e734–43. 10.1016/S2542-5196(24)00208-0. PMC11462510 39393375

[32] Bonjean CA, Brunelin S, Simonet C. (2012). Impact of climate related shocks on child’s health in Burkina Faso. FERDI Working Paper.

[33] Cebolla-Boado H, Suárez-Vergne Á, Martín TC, García TM. Birth weight in a warming world: investigating the protective role of the healthy immigrant effect against extreme heat. Popul Environ. 2025;47:13. 10.1007/s11111-025-00482-x.

[34] Connors JP, Casellas A, Safayet M, Rosenheim N, Watson M. Assessing changes in food pantry access after extreme events. Agric Hum Values. 2023;40:619–34. 10.1007/s10460-022-10373-8. PMC9616433 36340279

[35] Cooper MW, Brown ME, Hochrainer-Stigler S, Pflug G, McCallum I, Fritz S, Silva J, Zvoleff A. Mapping the effects of drought on child stunting. Proc Natl Acad Sci USA. 2019;116:17219–24. 10.1073/pnas.1905228116. PMC6717288 31405971

[36] Cooper M, Brown ME, Azzarri C, Meinzen-Dick R. Hunger, nutrition, and precipitation: evidence from Ghana and Bangladesh. Popul Environ. 2019;41:151–208. 10.1007/s11111-019-00323-8.

[37] Danysh HE, Gilman RH, Wells JC, Pan WK, Zaitchik B, Gonzálvez G, Alvarez M, Checkley W. El Niño adversely affected childhood stature and lean mass in Northern Peru. Clim Chang Responses. 2014;1:7. 10.1186/s40665-014-0007-z.

[38] Datar A, Liu J, Linnemayr S, Stecher C. The impact of natural disasters on child health and investments in Rural India. Soc Sci Med. 2013;76:83–91. 10.1016/j.socscimed.2012.10.008. PMC3544338 23159307

[39] Davenport F, Grace K, Funk C, Shukla S. Child health outcomes in Sub-Saharan Africa: a comparison of changes in climate and socio-economic factors. Glob Environ Change. 2017;46:72–87. 10.1016/j.gloenvcha.2017.04.009.

[40] del Ninno C, Lundberg M. Treading Water: The long-term impact of the 1998 flood on nutrition in Bangladesh. Econ Hum Biology. 2016;3:67–96. 10.1016/j.ehb.2004.12.002. 15722263

[41] Demeke AB, Keil A, Zeller M. Using panel data to estimate the effect of rainfall shocks on smallholders food security and vulnerability in rural Ethiopia. Clim Change. 2011;108:185–206. 10.1007/s10584-010-9994-3.

[42] Dimitrova A, Muttarak R. After the floods: differential impacts of rainfall anomalies on child stunting in India. Glob Environ Change. 2020;64:102130. 10.1016/j.gloenvcha.2020.102130.

[43] Dimitrova A, Bora JK. Monsoon weather and early childhood health in India. PLoS ONE. 2020;15:e0231479. 10.1371/journal.pone.0231479. PMC7147999 32275697

[44] Dimitrova A. Seasonal droughts and the risk of childhood undernutrition in Ethiopia. World Dev. 2021;141:105417. 10.1016/j.worlddev.2021.105417.

[45] Epstein A, Torres JM, Glymour MM, López-Carr D, Weiser SD. Do deviations from historical precipitation trends influence child nutrition? An analysis from Uganda. Am J Epidemiol. 2019;188:1953–60. 10.1093/aje/kwz179. PMC7415258 31497852

[46] Grace K, Davenport F, Funk C, Lerner AM. Child malnutrition and climate in Sub-Saharan Africa: An analysis of recent trends in Kenya. Appl Geogr. 2012;35:405–13. 10.1016/j.apgeog.2012.06.017.

[47] Grace K, Davenport F, Hanson H, Funk C, Shukla S. Linking climate change and health outcomes: examining the relationship between temperature, precipitation and birth weight in Africa. Glob Environ Change. 2015;35:125–37. 10.1016/j.gloenvcha.2015.06.010.

[48] Grace K, Verdin A, Dorélien A, Davenport F, Funk C, Husak G. Exploring strategies for investigating the mechanisms linking climate and individual-level child health outcomes: an analysis of birth weight in Mali. Demography. 2021;58:499–526. 10.1215/00703370-8977484. PMC8382135 33834220

[49] Hagos S, Lunde T, Mariam DH, Woldehanna T, Lindtjørn B. Climate change, crop production and child under nutrition in Ethiopia; a longitudinal panel study. BMC Public Health. 2014;14:884. 10.1186/1471-2458-14-884. PMC4158109 25163522

[50] Hanifi SMM, Menon N, Quisumbing A. The impact of climate change on children’s nutritional status in coastal Bangladesh. Soc Sci Med. 2022;294:114704. 10.1016/j.socscimed.2022.114704. 35030394

[51] Hirvonen K, Sohnesen TP, Bundervoet T. Impact of Ethiopia’s 2015 drought on child undernutrition. World Dev. 2020;131:104964. 10.1016/j.worlddev.2020.104964.

[52] Homma K, Md. Saiful Islam AH, Matsuura-Kannari M, Debela BL. Weather shocks and child nutritional status in rural Bangladesh: does labor allocation have a role to play? Food Policy. 2025;135:102918. 10.1016/j.foodpol.2025.102918.

[53] Hong Y. Heat and humidity on early-life outcomes: evidence from Mexico. J Environ Econ Manag. 2025;129:103082. 10.1016/j.jeem.2024.103082.

[54] Islam M, Ali S, Majeed H, Ali R, Ahmed I, Soofi S, Bhutta ZA. Drivers of stunting and wasting across serial cross-sectional household surveys of children under 2 years of age in Pakistan: potential contribution of ecological factors. Am J Clin Nutr. 2025;121:610–9. 10.1016/j.ajcnut.2025.01.003. 39788298

[55] Kamiya Y, Kishida T, Tanou M. Precipitation, temperature, and child undernutrition: evidence from the Mali demographic and health surveys 2012–2013 and 2018. J Health Popul Nutr. 2025;44:68. 10.1186/s41043-025-00808-3. PMC11887182 40050927

[56] Khan JR, Hossain MdB, Awan N. Community-level environmental characteristics predictive of childhood stunting in Bangladesh - a study based on the repeated cross-sectional surveys. Int J Environ Health Res. 2020;32:473–86. 10.1080/09603123.2020.1777947. 32602734

[57] LaPointe S, Mendola P, Lin S, Tian L, Bonell A, Adamba C, Palermo T. Impact of cash transfers on the association between prenatal exposures to high temperatures and low birthweight: retrospective analysis from the LEAP 1000 Study. BJOG. 2024;131:641–50. 10.1111/1471-0528.17761. 38238994

[58] Lazzaroni S, Wagner N. Misfortunes never come singly: structural change, multiple shocks and child malnutrition in rural Senegal. Econ Hum Biology. 2016;23:246–62. 10.1016/j.ehb.2016.10.006. 27794258

[59] Lopez-Carr D, Mwenda KM, Pricope NG, Kyriakidis PC, Jankowska MM, Weeks J, Funk C, Husak G, Michaelsen J. (2015). A spatial analysis of climate-related child malnutrition in the Lake Victoria Basin. In Proceedings of the 2015 IEEE International Geoscience and Remote Sensing Symposium (IGARSS), Milan, Italy, 2564–2567.

[60] López-Carr D, Mwenda KM, Pricope NG, Kyriakidis PC, Jankowska MM, Weeks J, Funk C, Husak G, Michaelsen J. Climate-related child undernutrition in the Lake Victoria basin: an integrated spatial analysis of health surveys, NDVI, and precipitation data. IEEE J Sel Top Appl Earth Observations Remote Sens. 2016;9:2830–5. 10.1109/JSTARS.2016.2569411.

[61] MacVicar S, Berrang-Ford L, Harper S, Huang Y, Namanya Bambaiha D, Yang S. Whether weather matters: evidence of association between in utero meteorological exposures and foetal growth among indigenous and non-indigenous mothers in rural Uganda. PLoS ONE. 2017;12:e0179010. 10.1371/journal.pone.0179010. PMC5462429 28591162

[62] Mank I, Belesova K, Bliefernicht J, Traoré I, Wilkinson P, Danquah I, Sauerborn R The impact of rainfall variability on diets and undernutrition of young children in rural Burkina Faso. Front Public Health. 2021;9:693281. 10.3389/fpubh.2021.693281. PMC8489680 34616704

[63] McMahon K, Gray C. Climate change, social vulnerability and child nutrition in South Asia. Glob Environ Change. 2021;71:102414. 10.1016/j.gloenvcha.2021.102414. PMC8653856 34898861

[64] Van Der Merwe E, Clance M, Yitbarek E. Climate change and child malnutrition: a Nigerian perspective. Food Policy. 2022;113:102281. 10.1016/j.foodpol.2022.102281.

[65] Molina O, Saldarriaga V. The Perils of Climate Change: In utero exposure to temperature variability and birth outcomes in the Andean region. Econ Hum Biology. 2017;24:111–24. 10.1016/j.ehb.2016.11.009. 27984771

[66] Ngwira A. Climate and location as determinants of childhood stunting, wasting, and overweight: an application of semiparametric multivariate probit model. Nutrition. 2020;70:100010. 10.1016/j.nutx.2020.100010. 34301371

[67] Nicholas K, Campbell L, Paul E, Skeltis G, Wang W, Gray C. Climate anomalies and childhood growth in Peru. Popul Environ. 2021;43:39–60. 10.1007/s11111-021-00376-8. PMC8389738 34456407

[68] Niles MT, Brown ME. A multi-country assessment of factors related to smallholder food security in varying rainfall conditions. Sci Rep. 2017;7:16277. 10.1038/s41598-017-16282-9. PMC5701123 29176578

[69] Omay PO, Bahaga TK, Fidar A. Insights into determinants influencing food security in the IGAD Region of Eastern Africa. Front Nutr. 2025;12:1600096. 10.3389/fnut.2025.1600096. PMC12202631 40584097

[70] Paul SK, Paul BK, Routray JK. Post-cyclone Sidr nutritional status of women and children in coastal Bangladesh: an empirical study. Nat Hazards. 2012;64:19–36. 10.1007/s11069-012-0223-4.

[71] Petscavage K, Mutua MK, Wagner AL, Treleaven E. Associations between extreme weather events and child undernutrition: evidence from Sub-Saharan Africa, 2010–2019. J Epidemiol Community Health. 2025;79:359–65. 10.1136/jech-2024-222748. 39694670

[72] Rahut DB, Mishra R, Bera S. Geospatial and environmental determinants of stunting, wasting, and underweight: empirical evidence from Rural South and Southeast Asia. Nutrition. 2024;120:112346. 10.1016/j.nut.2023.112346. 38320385

[73] Randell H, Gray C, Grace K. Stunted from the start: early life weather conditions and child undernutrition in Ethiopia. Soc Sci Med. 2020;261:113234. 10.1016/j.socscimed.2020.113234. PMC7716344 32823214

[74] Randell H, Jiang C, Liang XZ, Murtugudde R, Sapkota A. Food insecurity and compound environmental shocks in Nepal: implications for a changing climate. World Dev. 2021;145:105511. 10.1016/j.worlddev.2021.105511. PMC8128151 34012190

[75] Rodriguez-Llanes JM, Ranjan-Dash S, Degomme O, Mukhopadhyay A, Guha-Sapir D. Child malnutrition and recurrent flooding in Rural Eastern India: a community-based survey. BMJ Open. 2011;1:e000109. 10.1136/bmjopen-2011-000109. PMC3208901 22080535

[76] Rodriguez-Llanes J, Ranjan-Dash S, Mukhopadhyay A, Guha-Sapir D. Flood-Exposure Is Associated with Higher Prevalence of Child Undernutrition in Rural Eastern India. IJERPH. 2016;13:210. 10.3390/ijerph13020210. PMC4772230 26861372

[77] Rojas AJ, Gray CL, West CT. Measuring the Environmental Context of Child Growth in Burkina Faso. Popul Environ. 2023;45:3. 10.1007/s11111-023-00414-7. PMC10237046 37274602

[78] Rosinger AY, Rosinger K, Barnhart K, Todd M, Hamilton T, Arias Cuellar K, Nate D. When the Flood Passes, Does Health Return? A Short Panel Examining Water and Food Insecurity, Nutrition, and Disease after an Extreme Flood in Lowland Bolivia. Am J Hum Biol. 2023;35:e23806. 10.1002/ajhb.23806. PMC10116996 36165503

[79] Sam AS, Abbas A, Surendran Padmaja S, Kaechele H, Kumar R, Müller K. Linking Food Security with Household’s Adaptive Capacity and Drought Risk: Implications for Sustainable Rural Development. Soc Indic Res. 2019;142:363–85. 10.1007/s11205-018-1925-0.

[80] Sam AS, Abbas A, Surendran Padmaja S, Raghavan Sathyan A, Vijayan D, Kächele H, Kumar R, Müller K. Flood Vulnerability and Food Security in Eastern India: A Threat to the Achievement of the Sustainable Development Goals. Int J Disaster Risk Reduct. 2021;66:102589. 10.1016/j.ijdrr.2021.102589.

[81] Sandler A, Sun L. The socio-environmental determinants of childhood malnutrition: a spatial and hierarchical analysis. Nutrients. 2024;16:2014. 10.3390/nu16132014. PMC11243526 38999762

[82] Scharadin B, Zanocco C, Chistolini J. Food retail environments, extreme weather, and their overlap: exploratory analysis and recommendations for U.S. food policy. PLoS ONE. 2023;18:e0289282. 10.1371/journal.pone.0289282. PMC10631631 37939027

[83] da Silva LY, et al. Climate emergency and the food system: the impact of May floods on the community food environment of the Rio Grande do Sul State, Brazil. Cadernos de saúde pública. 2025;41:e00130824. 10.1590/0102-311XEN130824. PMC12108468 40435012

[84] Shaw S, Khan J, Paswan B. Spatial modeling of child malnutrition attributable to Drought in India. Int J Public Health. 2020;65:281–90. 10.1007/s00038-020-01353-y. 32240317

[85] Shively GE. Infrastructure mitigates the sensitivity of child growth to local agriculture and rainfall in Nepal and Uganda. Proc Natl Acad Sci USA. 2017;114:903–8. 10.1073/pnas.1524482114. PMC5293016 28096416

[86] Skoufias E, Vinha K. Climate variability and child height in rural Mexico. Econ Hum Biology. 2012;10:54–73. 10.1016/j.ehb.2011.06.001. 21840273

[87] Sultan B, Barriquault A, Brouillet A, Lavarenne J, Pongsiri M. Malnutrition and climate in Niger: findings from climate indices and crop yield simulations. IJERPH. 2025;22:551. 10.3390/ijerph22040551. PMC12026833 40283776

[88] Thiede BC, Gray C. Climate exposures and child undernutrition: evidence from Indonesia. Soc Sci Med. 2020;265:113298. 10.1016/j.socscimed.2020.113298. PMC7738425 32932006

[89] Thiede BC, Strube J. Climate variability and child nutrition: findings from Sub-Saharan Africa. Glob Environ Change. 2020;65:102192. 10.1016/j.gloenvcha.2020.102192. PMC8594912 34789965

[90] Tiwari S, Jacoby HG, Skoufias E. Monsoon babies: rainfall shocks and child nutrition in Nepal. Econ Dev Cult Change. 2017;65:167–88. 10.1086/689308.

[91] Tusting LS, Bradley J, Bhatt S, Gibson HS, Weiss DJ, Shenton FC, Lindsay SW. Environmental temperature and growth faltering in African children: a cross-sectional study. Lancet Planet Health. 2020;4:e116–23. 10.1016/S2542-5196(20)30037-1. PMC7232952 32220673

[92] Xiong X, Harville EW, Buekens P, Mattison DR, Elkind-Hirsch K, Pridjian G. Exposure to Hurricane Katrina, post-traumatic stress disorder and birth outcomes. Am J Med Sci. 2008;336:111–5. 10.1097/MAJ.0b013e318180f21c. PMC2635112 18703903

[93] Yeboah E, Kuunibe N, Mank I, Parisi D, Bonnet E, Lohmann J, Hamadou S, Picbougoum BT, Belesova K, Sauerborn R, et al. Every drop matters: combining population-based and satellite data to investigate the link between lifetime rainfall exposure and chronic undernutrition in children under five years in Rural Burkina Faso. Environ Res Lett. 2022;17:054027. 10.1088/1748-9326/ac661c.

[94] Yuan X, Zhou S, Li X, Lyu J, Lin L, Ji Y, Wang H, Liu J, Li Q, Wang H-J. The association of apparent temperature with fetal growth: a birth cohort study in Beijing, China. Sustainable Cities Soc. 2025;123:106266. 10.1016/j.scs.2025.106266.

[95] Mahapatra B, Walia M, Rao CAR, Raju BMK, Saggurti N Vulnerability of agriculture to climate change increases the risk of child malnutrition: evidence from a large-scale observational study in India. PLoS ONE. 2021;16:e0253637. 10.1371/journal.pone.0253637. PMC8238181 34181668

[96] Gomes SM, et al. Nexus among climate change, food systems, and human health: an interdisciplinary research framework in the Global South. Environ Sci Policy. 2024;161:103885. 10.1016/j.envsci.2024.103885.

[97] Saha J, Hussain D, Debsarma D. Exploring the association between floods and diarrhea among under-five children in rural India. Disaster Med Pub Health Prep. 2024;18:e142. 10.1017/dmp.2024.123. 39444209

[98] Moyo E, Nhari LG, Moyo P, Murewanhema G, Dzinamarira T. Health effects of climate change in Africa: A call for an improved implementation of prevention measures. Eco-Environment Health. 2023;2(2):74–8. 10.1016/j.eehl.2023.04.002. PMC10702879 38075293

[99] Hartinger S, Palmeiro-Silva Y, Llerena-Cayo C, et al. The 2023 Latin America report of the Lancet countdown on health and climate change: the imperative for health-centred climate-resilient development. Lancet Reg Health Am. 2024;31:100746. 10.1016/j.lana.2024.100746. PMC11117061 38800647

[100] Gie SM, McNeill G, Bannerman E. Retrofitting food and nutrition programmes to curb the global syndemic of undernutrition, obesity and climate change. Food Sect. 2024;16:1479–500. 10.1007/s12571-024-01489-2.

[101] IPCC. Climate Change 2022: Impacts, adaptation and vulnerability. Contribution of working group II to the sixth assessment report of the intergovernmental panel on climate change. Cambridge, UK and New York, NY, USA: Cambridge University Press; 2022.

