# Supplementary material for: Climate Events, Food Environment, and Nutritional Outcomes: A Scoping Review
Source: Curr Nutr Rep. 2026 Oct 29;15(1):90. doi: 10.1007/s13668-026-00809-6 (PMC13719856; doi:10.1007/s13668-026-00809-6)
Supplement: Supplementary file 1 [file 13668_2026_809_MOESM1_ESM.docx]

**Online Resource 1**

**Article "CLIMATE EVENTS, FOOD ENVIRONMENT, AND NUTRITIONAL OUTCOMES: A SCOPING REVIEW"**

**Table S1** Search strategy, databases, and retrieved references.

| **Search Strategy** | **Databases** | **Retrieved References** |
| --- | --- | --- |
| (((Weather, Extreme) OR (extreme weather) OR (climate change) OR (climate changes) OR (global warming) OR (warming climate) OR (Climate Change) OR (Extreme Weather Events) OR (Climate research)) AND ((obesity) OR (Body Weight) OR (Overweight) OR (Nutritional Status) OR (Status, Nutritional) OR (Status, Nutrition) OR (Malnutrition) OR (Nutritional deficiency) OR (Nutritional deficiencies) OR (undernutrition) OR (malnourishment) OR (malnourishments))) OR ((food environment) OR (food system))) | Scielo | 107 |
| ALL ( ( ( ( 'Weather, Extreme' OR 'extreme weather' OR 'climate change' OR 'climate changes' OR 'global warming' OR 'warming climate' OR 'Climate Change' OR 'Extreme Weather Events' OR 'Climate research' ) AND ( ( 'obesity' OR 'Body Weight' OR 'Overweight' ) OR ( 'Nutritional Status' OR 'Status, Nutritional' OR 'Status, Nutrition' ) OR ( 'Malnutrition' OR 'Nutritional deficiency' OR 'Nutritional deficiencies' OR 'undernutrition' OR 'malnourishment' OR 'malnourishments' ) ) ) OR ( 'food environment' OR 'food system' ) ) ) | Scopus | 4737 |
| ((((“Weather, Extreme” OR “extreme weather” OR “climate change” OR “climate changes” OR “global warming” OR “warming climate” OR “Climate Change” OR “Extreme Weather Events” OR “Climate research”)) AND ((“obesity” OR “Body Weight” OR “Overweight”) OR (“Nutritional Status” OR “Status, Nutritional” OR “Status, Nutrition”) OR (“Malnutrition” OR “Nutritional deficiency” OR “Nutritional deficiencies” OR “undernutrition” OR “malnourishment” OR “malnourishments”)))) OR ("food environment" OR "food system") | PubMed | 1914 |
|  | Web of Science | 2965 |

Source: Authors

**Table S2** Detailed methodological characteristics and mapping of the included studies in the review, by continent, climatic event, and outcome.

| **Author, year** | **Continent** | **Type of climatic event** | **Outcome** | | | **Methods** |
| --- | --- | --- | --- | --- | --- | --- |
|  |  |  | **Nutritional Status** | **Food Insecurity** | **Food Environmet** |  |
| Aguilar & Vicarelli, 2022 [19] | North America | Precipitation | Yes | No | No | - The study adopts a quasi-experimental design. Data from two main sources were combined for this study. One corresponds to the longitudinal database of the conditional cash transfer program evaluation (PROGRESA), collected semi-annually between 1997 and 2000 in marginalized rural communities in Mexico. In 2003, a follow-up survey collected information on children between 2 and 6 years of age. The other source corresponds to monthly precipitation data available from the Climatic Research Unit at the University of East Anglia (UEA CRU-TS2p1).  - From the anthropometric data, the following indicators were obtained: height-for-age z-score, weight-for-age z-score, and stunting (height-for-age < -2 z-score).  - Based on monthly precipitation data, the Standardized Precipitation Index was created, obtained by calculating monthly precipitation anomalies in standard deviations relative to the historical mean (1961–1999). Precipitation extremes were defined as the occurrence of SPI > 0.7 during the months of September or October 1999, coinciding with the harvest season. The effect of exposure to precipitation extremes was assessed during the in utero phase or the first two years of the child's life.  - Analyses were conducted by comparing birth cohorts exposed and unexposed to the anomalies. |
| Ahmad et al, 2022 [20] | Asia | Precipitation and Floods | No | Yes | No | - Study conducted with 384 rural households between September and November 2019 in the flood-prone areas of the Bait community, in Punjab, Pakistan.  - Household food security was assessed using an index based on 11 indicators organized according to the four dimensions established by the FAO (access, availability, utilization, and stability). These indicators were combined using polychoric principal component analysis.  - Perception of flood-related factors was assessed: property losses due to floods (whether families experienced losses of property, such as durable goods, housing, or livestock, during the 2010–2015 flood period); presence of flood-induced stress; average precipitation variation (whether the family reported any variation in mean precipitation from 2010 to 2015); and income affected by environmental degradation (whether household income was affected due to environmental degradation caused by climate change and floods). |
| Ahmed et al, 2021 [21] | Africa | Temperature, Precipitation, and Aridity | Yes | No | No | - The study used data from the 2016 Ethiopia Demographic and Health Survey, involving 9,089 children aged 0 to 59 months residing in 622 clusters. For each cluster, climate data extracted from publicly available raster and vector remote sensing sources were linked.  - For each cluster, the prevalence of stunting (height-for-age < -2 z-score) was calculated.  - The climate variables assessed were: daytime land surface temperature (<30°C; 30–34.99°C; ≥35°C); mean annual precipitation (<141 mm; 142–1199 mm; ≥1200 mm); aridity (defined as the mean annual precipitation divided by the mean annual potential evapotranspiration, categorized as "arid" if the index was <17.5, "semi-arid" if between 17.5 and 32.5, and "humid" if >32.5); and the number of rainy days per year (categorized as: "low" if <6 days, "medium" if 6–8 days, and "high" if >8 days). |
| Ahsanuzzaman &Islam, 2020 [22] | Asia | Cyclone | Yes | No | No | - The study used data on children aged 0 to 59 months residing in households included in the 2011 Bangladesh Demographic and Health Survey. Based on the residential addresses, it was determined whether the children lived in areas affected or unaffected by Cyclones Sidr (November 2007) and Aila (May 2009).  - From the children's weight and height data, the following anthropometric indicators were obtained: height-for-age, weight-for-age, and weight-for-height z-scores. These variables were also dichotomized into: stunting (height-for-age < -2 z-score), underweight (weight-for-age < -2 z-score), and wasting (weight-for-height < -2 z-score).  - Information on the month of birth allowed for determining whether the child was exposed to the event during pregnancy, at birth, or after the first months of life. Children were divided into three groups according to the timing of cyclone exposure: children (up to 12 months old at the time of the event) born before Sidr and Aila (neonatal exposure), children exposed in utero (whose mothers were pregnant during the cyclone, separated by trimester), and a pre-utero group (whose mothers conceived after the cyclones).  - A difference-in-differences (estimation technique was used to compare the outcomes of children from two different regions, before and after the event, to artificially construct a counterfactual. The larger administrative unit (division) was used as the location unit to create treated and untreated samples. |
| Alterman et al, 2024 [23] | Asia | Temperature | Yes | No | No | - This is a population-based historical cohort study, including 1,100,576 infants born between 2011 and 2019, sourced from the Israel National Network of Maternal and Child Health Clinics.  - Repeated weight measurements between birth and 15 months were used to estimate weight gain velocity using the Superimposition by Translation and Rotation (SITAR) method. Rapid infant weight gain was determined, defined as the top tertile of the SITAR growth velocity.  - Daily ambient temperature measurements were assessed using a hybrid model that generates daily mean temperature estimates at a 1 × 1 km resolution, utilizing satellite remote sensing data validated against meteorological stations. The families' residential addresses at birth were geocoded. The mean temperature at those locations was calculated for several periods: each pregnancy trimester (gestational weeks 1–13, 14–26, and 27–delivery) and for the first postnatal month. Weekly averages were also calculated considering these prenatal and postnatal periods. Temperature estimates were categorized into quintiles (<15.4, 15.4–19.4, 19.4–23.6, 23.6–26.7, and >26.7°C). |
| Alterman et al, 2025 [24] | Asia | Temperature | Yes | No | No | - This is a population-based historical cohort study using data from the national network of maternal and child health clinics (968,534 infants included in the 1-year-old analyses, of whom 623,890 were assessed at 2 years of age).  - Based on weight and height data, overweight was determined using the weight-for-height z-score (WHZ) at 1 year of age and the Body Mass Index-for-age (BMI-for-age) at 2 years of age.  - Ambient temperature was assessed using a spatiotemporal hybrid prediction model with a 1 × 1 km resolution, which combines satellite sensor data, temperature monitoring, land use, and atmospheric data. Daily mean and minimum temperatures were estimated using the family's most recent residential address for geocoding. Weekly averages of these temperatures were calculated for gestational weeks 0 to 36 and during the first year of life, with measurements subsequently categorized into quintiles. |
| Amegbor et al, 2025 [25] | Africa | Temperature and Precipitation | Yes | No | No | - Data from 343,199 children under 5 years of age from 30 Sub-Saharan African countries included in the Demographic and Health Surveys (2004–2021) were combined with temperature and precipitation data obtained from the Climatic Research Unit gridded Time Series (CRU TS) for the period from 1901 to 2022.  - Based on weight and height, stunting (height-for-age < -2 z-score) was determined.  - Standardized anomalies of mean and maximum precipitation and temperature values were obtained for the prenatal period (9 months before birth) and postnatal period (first 12 months of life). To achieve this, the average values of these climate data were calculated for the assessed period (prenatal or postnatal). These values were then standardized by comparing them to historical 9-month periods in the climate datasets for prenatal anomalies and 12-month periods for postnatal anomalies. |
| Ampofo et al, 2025 [26] | Oceania | Temperature | Yes | No | No | - Data were used from Australian adults in the Household, Income and Labour Dynamics in Australia (HILDA) longitudinal survey, covering the period from 2006 to 2022. Daily temperature and precipitation data at the household postcode level were based on ERA5 satellite reanalysis data from the European Centre for Medium-Range Weather Forecasts (ECMWF).  - Weight and height data were self-reported, from which the Body Mass Index (BMI) was calculated, and individuals were classified as having or not having obesity.  - Temperature bins were constructed for days between 2006 and 2022 below 5°C and above 30°C, representing the number of days an individual is exposed to a mean temperature range within a postcode. |
| Baez et al, 2024 [27] | Africa | Drought | Yes | No | No | - Sociodemographic and anthropometric data of children under 5 years of age (living in rural areas) from Demographic and Health Surveys in five Southern African countries (Malawi, Tanzania, Mozambique, Zimbabwe, and Zambia) were combined with climate data (2010 to 2019).  - The outcome was derived from the children's anthropometric data to calculate the height-for-age z-score indicator.  - The explanatory variables were constructed using precipitation data from the Climate Hazards Group InfraRed Precipitation with Station and the Normalized Difference Vegetation Index, both obtained via remote sensing. The primary exposure, drought occurrence, was defined as a sequence of three or more consecutive 10-day periods (dekads) of standardized precipitation below the mean during the crop growing season (using the vegetation index to estimate the harvest cycle). Previous drought exposure was assessed at two key moments: pre-birth drought (if the harvest cycle prior to birth was disrupted) and next-season drought (if the harvest cycle following birth was disrupted). |
| Baffour et al, 2023 [28] | Asia | Aridity, Temperature, Precipitation | Yes | No | No | - The study used cluster-level data from the Demographic and Health Surveys for Bangladesh (2017–2018) and Ghana (2014). Climate data were integrated via remote sensing from the Center for Environmental Data Analysis Web Processing Service for the assessed clusters.  - The outcome variable used was the prevalence of stunting (defined as height-for-age below -2 z-scores) in children under 5 years of age.  - Aridity was calculated as the ratio between mean monthly precipitation and mean monthly potential evapotranspiration. Annual precipitation was calculated by summing monthly precipitation plots. Mean annual temperature (in degrees Celsius) was derived from monthly near-surface temperature data for 2015. Exposure was assessed considering a 2 km buffer for urban clusters and a 5 km buffer for rural clusters around each survey cluster. Bayesian geostatistical models were applied to estimate the prevalence of the outcome. |
| Baker & Anttila-Hughes, 2020 [29] | Africa | Temperature | Yes | No | No | - This is an observational quantitative study with cross-sectional and longitudinal analysis. Anthropometric data from approximately 190,000 children aged 1 to 5 years, extracted from Demographic and Health Surveys (conducted between 1993 and 2012 in 28 Sub-Saharan African countries), were integrated with mean monthly temperature data obtained from the University of Delaware Climate Data (0.5° x 0.5° resolution).  - Standardized indicators for height-for-age, weight-for-age, and weight-for-height z-scores were obtained from the anthropometric data.  - The explanatory variable was the mean monthly temperature. Climate measurements were linked based on the location and date of the interview, considering three time scales: mean temperature in the month of the interview, mean temperature in the preceding 12 months, and lifetime mean temperature of the child. Additionally, the mean temperature during the predominant growing season (maize, wheat, or rice) was analyzed based on geospatial crop data. |
| Basu et al, 2018 [30] | North America | Temperature | Yes | No | No | - This is a retrospective cohort study with information obtained from the California Department of Public Health’s Center for Health Statistics and Informatics Birth Data File, covering the period between 1999 and 2013. The cohort included 43,629 infants with low birth weight and 2,032,601 infants with normal weight, all of whom were full-term births (37 to 44 gestational weeks). Climate data were obtained from three sources: California Irrigation Management Information System, US Environmental Protection Agency, and National Centers for Environmental Information.  - Low birth weight (< 2,500 grams) among full-term births was defined based on weight and gestational age records available on birth certificates.  - Daily apparent temperature (a combination of temperature and relative humidity, in degrees Fahrenheit) was calculated, and the explanatory variable was the weekly mean apparent temperature, analyzed continuously in 10ºF increments. The exposure periods considered were: full gestation, gestational trimesters, the first month of pregnancy, the month prior to delivery, and the two weeks prior to delivery. Exposure was assigned based on the proximity of the maternal residential address to weather stations (a radius of up to 10 km within specific climate zones). Cut-off points were defined for better model fit, according to dose-response curves (55ºF for full gestation and 60ºF for the first trimester). |
| Blom et al, 2022 [31] | Africa | Heatwave | Yes | No | No | - The study used sociodemographic and anthropometric data of children aged 3 to 36 months from 15 rounds of Demographic and Health Surveys (conducted between 1993 and 2014) in five West African countries (Benin, Burkina Faso, Ivory Coast, Ghana, and Togo). Georeferenced climate data (temperature and precipitation) were obtained from the Global Meteorological Forcing Dataset, which provides daily near-surface temperature and precipitation data.  - Standardized height-for-age and weight-for-height z-score indicators were generated. Additionally, dichotomous variables were assessed for the presence of stunting (defined as height-for-age below -2 z-scores) and wasting (defined as weight-for-height below -2 z-scores).  - The climate explanatory variables were temperature and precipitation. Heat exposure was measured as the average number of hours per month in different temperature bins (<30ºC; 30 to 35ºC; >35ºC). Lifetime exposure was used for the height-for-age indicator, while exposure in the last 90 days was used for the weight-for-height indicator. Climate data were linked to the household cluster-level data from the Demographic and Health Surveys. |
| Bonell et al, 2024 [32] | Africa | Heat stress | Yes | No | No | - This is a longitudinal study derived from the Early Nutrition and Immunity Development randomized clinical trial, conducted in West Kiang, The Gambia (January 20, 2010, to February 10, 2015). The trial assessed the impact of nutritional supplementation during the first 1,000 days of life. Climate data for heat stress were obtained from the Human Thermal Comfort dataset of the Copernicus Climate Change Service.  - Various anthropometric indicators were assessed at two primary time points: prenatal, including length-for-gestational-age, weight-for-gestational-age, and head-circumference-for-gestational-age z-scores; and postnatal (at 2 years of age), using weight-for-height, weight-for-age, and height-for-age z-scores.  - Exposure to heat stress was estimated using the Universal Thermal Climate Index (a composite measure of temperature, humidity, wind speed, and solar radiation) obtained from the Human Thermal Comfort dataset, specific to the location of the children's residences. Heat stress was defined as minimum, mean, maximum, or daytime daily exposure, with the mean calculated per gestational trimester. |
| Bonjean et al, 2012 [33] | Africa | Precipitation | Yes | No | No | - The study combined data from the National Survey on Food Insecurity and Malnutrition, conducted between June and July 2008, with daily rainfall data from 138 meteorological stations in Burkina Faso (1995–2008). The target population included 12,571 children under 5 years of age living in rural areas of Burkina Faso.  - The nutritional outcome indicator used was the height-for-age z-score, derived from the children's anthropometric data.  - The primary explanatory variable was precipitation, summed during the crop growing season (June to September). The indicator was calculated as the percentage difference between the annual precipitation and the 1995–2008 mean. Precipitation extremes (positive and negative) were also assessed, classified by magnitude (small/large) using standard deviation. Exposure was evaluated across several periods: the in utero period and the first months of life, and the first and second years of life, using data from the nearest weather station to each municipality (or the second nearest, if there were data gaps). |
| Cebolla-Boado et al, 2025 [34] | Europe | Temperature | Yes | No |  | -This cross-sectional study utilized birth data—including birth weight, prematurity, macrosomia, and low birth weight—along with maternal health and sociodemographic characteristics, obtained from the National Statistics Institute of Spain. Temperature data were collected from the State Meteorological Agency.  - The outcome variables included low birth weight, prematurity, macrosomia, and birth weight.  - The explanatory variable was the mean daily temperature, aggregated by administrative regions and estimated for daily values, categorized into Celsius degree intervals. Temporal exposure considered temperatures corresponding to specific gestational weeks. For the analysis, linear regression models were employed to estimate mean effects of temperatures, and quantile regressions were used to explore heterogeneities in the effects at different points of the outcome distributions. Additionally, marginal effects tests were performed to evaluate the impact in different contexts, controlling for confounding variables such as maternal age, education level, marital status, and housing conditions. |
| Connors et al, 2022 [35] | North America | Floods | No | No |  | - The study utilized geospatial data collection from various sources, such as the United States Census Bureau, the Federal Emergency Management Agency, and the Houston Food Bank, to map the locations of food distribution sites, flood-risk areas, sociodemographic characteristics, and the road network of Harris County.  - The outcome variable was the level of food access (classified as high, medium, or low), as well as the proportion of census blocks that changed access categories due to road network disruptions.  - The exposure was the road network disruption caused by flooding events. Geographic Information Systems were used to model the road network and identify accessible and disrupted routes, considering infrastructural elements (elevated or underground streets). Buffers of 1 to 5 miles were used around distribution sites, with 1 mile representing an urban standard and 5 miles reflecting typical travel distances in the region. The primary analysis consisted of comparing the number of census blocks at different food access levels under baseline and disruption scenarios. Factors such as flood duration and the effects on the operational capacity of food assistance networks were considered. A sensitivity analysis was also performed by varying the buffer size from 1 to 5 miles. |
| Cooper et al, 2019 [36] | South America, Africa, Asia and Oceania | Drought | Yes | No | No | - The study utilized a combination of sociodemographic and nutritional data from Demographic and Health Surveys (127 surveys conducted across 53 countries, 1990–2016), covering 584,662 children aged 0–59 months living in predominantly agricultural regions. Climatological data and global geographic datasets were also used from various sources, such as the World Bank, the Food and Agriculture Organization, NASA, and datasets published in scientific journals regarding factors influencing food system sensitivity and local adaptive capacity.  - The height-for-age z-score was the indicator used.  - The primary exposure was measured by the Standardized Precipitation-Evapotranspiration Index, which assesses variations in hydrological conditions. This index was calculated by taking into account both precipitation and evapotranspiration. The index was categorized into: drought (index less than -0.4), normal precipitation periods (between -0.4 and 1.4), and precipitation extremes (greater than 1.4). The index was calculated considering 12, 24, and 36-month windows, as well as the child's entire lifetime, including time in utero. |
| Cooper et al, 2019 [37] | Africa and Asia | Precipitation | Yes | Yes | No | - The study used data from the Feed the Future household surveys conducted in Ghana (2012) and Bangladesh (2011 and 2015). The study population included 1,346 children from Ghana and 3,271 from Bangladesh, all under 5 years of age, and their respective households located in rural areas of both countries. Climate data were obtained from the Climate Hazards Group Infra Red Precipitation with Station database.  - The outcome variables were: height-for-age and weight-for-height z-scores (indicators of chronic and acute child malnutrition, respectively), and the Household Hunger Scale —a household food insecurity indicator composed of three standardized questions.  - Precipitation was operationalized using the Standardized Precipitation Index, a measure derived from the precipitation variation for a given location based on long-term norms. Positive values indicate periods wetter than average, while negative values indicate drier periods. The index was calculated across 12, 24, 36, 48, and 60-month windows. |
| Danysh et al, 2014 [38] | South America | Floods | Yes | No | No | - This is a cross-sectional study conducted in 2008 and 2009. The sample included 2,095 children from 59 rural villages, randomly selected based on birth year (1991–2001). Data were collected in November (dry season) and December (rainy season) in 2008 and 2009 to account for seasonality.  - The outcome variables included height-for-age z-scores and estimates of fat mass and lean mass in kilograms (obtained via bioelectrical impedance for 912 children).  - The primary exposure was the flood probability score, representing the probability of a child living in a flood-prone residence between 1991 and 2001. This index was calculated in two steps using residential coordinates, soil saturation percentage estimates generated from simulations with the Noah Land Surface Model, and meteorological data from NASA’s Modern-Era Retrospective analysis for Research and Applications. First, a generalized linear mixed model (GLMM) with an identity link function was used to estimate smoothed soil moisture values; flooding was defined as a studentized residual greater than 2 (soil saturation exceeding 97.5%). Second, a GLMM with a logit link function calculated the predicted probability that a child lived in a flood-prone location (flood probability score), considering the number of months since birth the residence was classified as flooded. Exposure was also assessed relative to the 1997–1998 El Niño through two birth-date indicators (the number of years between birth and January 1991, and the number of years between birth and the onset of El Niño for children born afterward). |
| Datar et al, 2013 [39] | Asia | Drought, Temperature, Floods, Storm | Yes | No | No | - The study combined data from children under 5 years of age residing in rural households, included in the National Family Health Survey of India (conducted in 1992–93, 1998–99, and 2005–06), with disaster data (drought, flooding, storms, mass movement, and earthquakes) recorded in the Emergency Events Database for events occurring in India during the periods of 1991–93, 1997–99, and 2004–06.  - The outcome variables were standardized anthropometric indicators—height-for-age, weight-for-age, and weight-for-height z-scores, along with their respective dichotomous categorizations (stunting, underweight, and wasting).  - The primary exposure was to disasters (drought, flooding, storms, mass movement, and earthquakes). The exposure variable was coded as a dummy indicator, assigned a value of 1 if the child was exposed to any disaster. Disaster exposure was defined based on the eleven months prior to the interview date, including the month of the interview. |
| Davenport et al, 2017 [40] | África | Precipitation and Temperature | Yes | No | No | - The study used a combination of household-based Demographic and Health Survey (DHS) data, covering the period from 1997 to 2010, and country-level data on socioeconomic trends, alongside gridded estimates of precipitation and temperature.  - The outcome variables were standardized anthropometric indicators: birth weight (in grams) and height-for-age z-score (HAZ) for children under 5 years of age.  - The climate explanatory variables were precipitation and daily maximum temperature. Gridded precipitation data were used from the Climate Hazards Group InfraRed Precipitation with Station (CHIRPS) (0.5° resolution, ~5 km). Precipitation records for each child were generated by averaging the grid cells within a 10 km radius around the sampling cluster. Daily maximum temperature data were sourced from a global gridded dataset (0.5° x 0.5° resolution). The mean daily maximum temperature was calculated within a 10 km radius around the associated sampling cluster. |
| Dell Ninno & Lundberg, 2005 [41] | Asia | Floods | Yes | No | No | - The study was developed using data from children up to 60 months of age (499 children interviewed, 237 with complete data) residing in households included in a three-stage household survey, part of the Food Management and Research Support Project (supported by the World Bank) across seven administrative divisions of Bangladesh affected by the 1998 floods. Data were collected between November 1998 and November 1999; the first stage took place 2 months after the flood, and the third stage 15 months later.  - The outcome variables were standardized height-for-age and weight-for-height indicators, derived from anthropometric weight and height data.  - The primary exposure was direct flood exposure, which was assessed through a household flood exposure index. This index is a continuous variable ranging from 0 to 16, constructed based on water depth on the property and in the house, as well as the duration (number of days) of water inside the home. Households were classified as unexposed (values equal to zero) or flood-exposed (values greater than or equal to 1). The longitudinal design allowed for the assessment of effects at three time points following the flood (2 months, April 1999, and 15 months). |
| Demeke et al, 2011 [42] | Africa | Precipitation | No | Yes | No | - The study utilized three rounds of the Ethiopian Rural Household Survey dataset (1994, 1999, and 2004). The survey included 1,477 rural households from 15 villages, representing diverse farming systems across the country.  - A relative household food security index was constructed using Principal Component Analysis (PCA) based on five indicators: cultivated land size, existence of food stocks, food group diversity consumed, variety of crops planted, and ox ownership. Based on the index’s evolution over time, relative household food security was categorized as: persistently insecure (negative index in all rounds); vulnerable (fluctuating between positive and negative index); and persistently secure (positive index in all rounds).  - The study utilized both subjective and objective measures of precipitation. A perception-based rainfall index was constructed from a set of questions regarding the timeliness, amount, and distribution of rainfall. Objective measures included mean annual precipitation (in millimeters) obtained from a weather station near the village, and the rainfall deviation from the 30-year historical mean (in millimeters). |
| Dimitrova & Muttarak, 2020 [43] | Asia | Precipitation | Yes | No | No | - The study used health data obtained from the 2015–2016 Demographic and Health Survey. The final sample included 220,823 children under five years of age. Gridded rainfall data were obtained from the Climatic Research Unit at the University of East Anglia.  - From the anthropometric data, the standardized height-for-age z-score was derived, along with dichotomous variables for stunting (height-for-age < -2 z-score) and severe stunting (height-for-age < -3 z-score).  - The total amount of monsoon season precipitation in millimeters was calculated for each location. The primary measure constructed was precipitation anomalies, defined as deviations in monsoon season rainfall from the long-term mean (1970 to 2016). Positive anomalies indicated excess rainfall, while negative anomalies indicated scarcity. Mean precipitation anomalies were measured from the in utero period up to the time of the interview. |
| Dimitrova, Bora, 2020 [44] | Asia | Precipitation | Yes | No | No | - The study combined anthropometric data of children aged 0–59 months and household sociodemographic data from the 2015–2016 India Demographic and Health Survey with georeferenced climate data. Data for the precipitation extremes indicator were sourced from the Climatic Research Unit time series.  - From the anthropometric data, standardized height-for-age and weight-for-height z-scores were derived. The dichotomous variables classified were: stunting (< -2) and severe stunting (< -3); and wasting (< -2) and severe wasting (< -3).  - The primary exposure was measured by the Standardized Precipitation-Evapotranspiration Index, which measures monthly variations in net precipitation minus potential evapotranspiration due to temperature, compared to the site-specific long-term mean. The index values were categorized into: drought events (≤ -1.5) and excessive rainfall events (≥ 1.5). Climate data means were calculated within a 10 km buffer around the household clusters, using monthly data during the summer (monsoon months of June to September). For stunting (chronic effect), exposure during the in utero period and the first year of life was considered. For wasting (acute effect), exposure to the index during the last monsoon season prior to the child's measurement was considered. |
| Dimitrova, 2021 [45] | Africa | Drought | Yes | No | No | - The study utilized anthropometric data from 21,551 children under five years of age and household sociodemographic data, included in three rounds of the Ethiopia Demographic and Health Surveys (2005, 2011, and 2016). Monthly climate data for precipitation and evapotranspiration were obtained from the Climatic Research Unit at the University of East Anglia.  - The nutritional status of the children was classified based on standardized anthropometric indicators for the presence of stunting and wasting.  - Drought was measured using the Standardized Precipitation-Evapotranspiration Index, calculated at 4-month and 12-month scales (for seasonal and agricultural droughts, respectively). Drought was defined as an índex value less than or equal to -1.25. Climate data means were calculated within a 10 km buffer around the household clusters. To assess stunting (chronic outcome), exposure was defined as the average index values over the child's observed lifetime (from birth to measurement), calculated by season (belg, meher, and bega). For wasting (acute outcome), the index values during the most recent belg, meher, and bega seasons prior to the interview were considered. |
| Drysdale et al, 2020 [3] | Africa | Precipitation | No | Yes | No | -The study employed a comparative study design, consistent with a natural experiment, by repeating two cross-sectional household surveys (Round 1: 2016; Round 2: 2017) in the iLembe District Municipality, KwaDukuza, South Africa. Precipitation data were obtained from the South African Weather Service.  - Food insecurity scores were obtained using the Coping Strategies Index.  - The exposure was severe drought (Round 1) followed by a period of increased average precipitation (Round 2), characterizing the natural experiment. Annual precipitation was calculated for each sub-district using data from the South African Weather Service. Round 1 used data from November 2015 to October 2016, and Round 2 used data from November 2016 to October 2017. The study compared the results obtained during the severe drought (Round 1) and after the increase in precipitation (Round 2). |
| Epstein et al, 2019 [46] | Africa | Drought and Precipitation | Yes | No | No | - The study utilized sociodemographic household data and anthropometric data of children participating in the Uganda National Panel Survey 2009–2012, a representative longitudinal panel with repeated measures in children aged 6 to 59 months.  - The outcome variables were the repeated measures in children aged 6 to 59 months, including weight-for-age, height-for-age, and weight-for-height z-scores, as well as underweight, stunting, and wasting.  - The study utilized both subjective and objective measures of climate exposure. The subjective measure was the household head's perception of drought or irregular rainfall ("Did you suffer from drought/irregular rainfall in the last 12 months?", yes/no). The objective measure was cumulative precipitation obtained from the Climate Hazards Group InfraRed Precipitation with Station database. A continuous value for monthly precipitation at each household's GPS location was extracted and summed for the preceding 12 months. The cumulative precipitation deviation was calculated as the difference between this value and the mean annual precipitation of the last 10 years (1999 to 2008). |
| Grace et al, 2012 [47] | Africa | Temperature and Precipitation | Yes | No | No | - The study was developed using data from 1986 to 2010, covering 67,769 newborns residing in households included in the Demographic and Health Surveys across 19 African countries. Temperature and precipitation data were derived from combined satellite observations and ground stations.  - The dependent variables were birth weight and the categorical measure of low birth weight, using the World Health Organization cut-off point of 2,500 grams (yes/no).  - Environmental data (precipitation, measured from high-spatial-resolution monthly data, and temperature) were linked to the child's in utero experience (trimesters 0 to 3) using the birth date and the sampling cluster location within a 10 km radius for a one-year period prior to birth. Precipitation exposure was assessed as quarterly precipitation and during the agricultural growing season. Temperature exposure was constructed based on the count of hot days per trimester, defined as those where the daily maximum temperature exceeded specific thresholds for arid environments (40.9°C) and less hot environments (38.7°C), as well as during the agricultural growing season. |
| Grace et al, 2015 [48] | Africa | Precipitation and Temperature | Yes | No | No | - The study combined household socioeconomic data and anthropometric measures of children aged 1 to 5 years, obtained from the 2008 Kenya Demographic and Health Survey (DHS), with climate data from 1998 to 2008. The climate data were interpolated from a combination of ground-based weather stations, remote sensing data, and a priori knowledge of climate patterns.  - The outcome variables were anthropometric measures for children aged 1 to 5 years, specifically height-for-age z-scores.  - The climate explanatory variables were precipitation levels (mean total precipitation in millimeters for each year of the child's life), precipitation variation, land surface temperature (mean over the child's lifetime), and temperature variation. Climate data (precipitation and temperature) were calculated using March-to-June season means for the period assessed, linked to households based on sampling cluster locations. Values were calculated across grid cells within a 13 km buffer around each cluster. The analysis also incorporated data on population density to differentiate rural from metropolitan regions, and climate data interpolation was used to capture spatial trends and smooth local variations. |
| Grace et al, 2021 [49] | Africa | Temperature and Precipitation | Yes | No | No | - The study utilized a database from three rounds of cross-sectional surveys (2000, 2006, and 2012) conducted in Mali through the Demographic and Health Surveys (DHS). The study population consisted of 9,542 newborns residing in the municipalities included in the survey. Heat stress data were developed by Princeton University, and precipitation data were obtained from the Climate Hazards Center InfraRed Precipitation with Station (CHIRPS).  - Birth weight was assessed both continuously and categorically (low birth weight: less than 2,500 grams).  - Heat stress, derived from Princeton University meteorological data, was calculated as the number of days with a maximum temperature above 100°F during the trimesters of pregnancy. The mean monthly maximum temperature during these trimesters was also used. The mean monthly precipitation in each gestational trimester was calculated from data obtained from CHIRPS. The exposure period evaluated was: Trimester 0 (three months prior to conception), 1st gestational trimester, 2nd gestational trimester, and 3rd gestational trimester. |
| Hagos et al, 2014 [50] | Africa | Precipitation and Temperature | Yes | No | No | - The longitudinal panel study used data obtained from Demographic and Health Surveys conducted between 1996 and 2004, covering 145 children under 5 years of age across 43 administrative zones in Ethiopia. Precipitation data were provided by the Malaria Prediction System Research Project and the National Meteorological Authority, while temperature data were obtained from the Climatic Research Unit time-series datasets.  - The nutritional outcome indicators derived from the children's weight and height data were: stunting (height-for-age below -2 z-scores), severe stunting (height-for-age below -3 z-scores), underweight (weight-for-age below -2 z-scores), severe underweight (weight-for-age below -3 z-scores), wasting (weight-for-height below -2 z-scores), and severe wasting (weight-for-height below -3 z-scores).  - The explanatory variables were precipitation and temperature, compiled for the years 1996, 1998, 2000, and 2004 for each study zone. Precipitation exposure was measured using mean monthly rainfall data for the months of June to September (the primary harvest season); precipitation anomalies were calculated to generate standardized precipitation data. The analysis assessed the association of linear and quadratic terms for standardized precipitation and mean temperature (in degrees Celsius) with the nutritional outcomes. |
| Hanifi et al, 2022 [51] | Asia | Precipitation, Humidity, Temperature | Yes | No | No | - The study utilized sociodemographic and anthropometric data from 19,357 children under 3 years of age (from 13,197 households) born between January 2011 and March 2020, residing in households assessed by the Demographic and Health Surveillance System across 49 villages in the Chakaria sub-region of Bangladesh. Climate data were obtained from the Bangladesh Meteorological Department’s weather station located in Cox’s Bazar.  - The anthropometric outcome variable assessed in the children was the mid-upper arm circumference (measured in millimeters).  - Temperature was assessed in 5°C intervals, and both temperature and humidity were evaluated using linear and quadratic forms. These climate data were linked to the child’s sample data by month and year of birth. The analysis considered exposure to climate events during the month and year of conception, throughout the entire intrauterine period, and by gestational trimesters. |
| Hirvonen et al, 2020 [52] | Africa | Drought | Yes | No | No | - The study employed a quantitative approach using a difference-in-differences design, utilizing longitudinal data from the two most recent rounds of the Ethiopian Socioeconomic Survey (2014, pre-drought; and 2016, post-drought). The analysis was restricted to rural areas of the country and included children aged 6 to 59 months (n=1,874 in 2014 and n=1,708 in 2016). Precipitation climate data were obtained from the Climate Hazards Group InfraRed Precipitation with Station database.  - From the anthropometric data, standardized indicators for height-for-age and weight-for-height z-scores were derived, as well as dichotomous indicators for stunting (height-for-age below -2 z-scores) and wasting (weight-for-height below -2 z-scores).  - The primary exposure was drought, measured by precipitation. Climate data were linked to the household's latitude and longitude coordinates using an inverse distance weighted average of the four nearest satellite observations. Precipitation z-score deviations were calculated during the meher season (June to September) relative to the long-term mean (2000–2015) in the same location. Areas exposed to drought were defined as those where annual precipitation during the meher season fell below 2 standard deviations. |
| Homma et al, 2025 [53] | Asia | Precipitation and Temperature | Yes | No | No | - The study is based on data from the Bangladesh National Survey. Climate data were derived from historical precipitation and temperature records.  - Nutritional data collected across various survey waves were categorized using weight-for-age z-scores.  - The climate explanatory variables were based on precipitation and temperature anomalies in the year of the survey and the year prior to the survey. Climate data were aggregated at the regional level, linked to agricultural activities and environmental conditions of the period studied. |
| Hong, 2025 [54] | North America | Temperature and Humidity | Yes | No | No | - The study adopted a quasi-experimental approach, using administrative data from birth and stillbirth records in Mexico, obtained from official registries and covering the period from 2008 to 2021. These data were combined with meteorological temperature and humidity data obtained from nearby stations.  - The variables for birth weight, birth length (in centimeters), and the probability of birth weight below 2.5 kg were obtained from official records.  - The primary exposure was the wet-bulb temperature, calculated using the Stull method, which integrates dry temperature and relative humidity. To capture the duration of exposure, the number of days per month in which the wet-bulb temperature exceeded certain thresholds was considered, with a focus on intrauterine exposure, particularly during the last trimester. |
| Islam et al, 2025 [55] | Asia | Precipitation and Temperature | Yes | No | No | - This is a cross-sectional study based on two rounds of the Pakistan National Survey, conducted in 2011 and 2018. The sample included 29,887 children aged 0 to 23 months, covering 140 districts across the country. Mean temperature and precipitation data were acquired from a high-resolution gridded dataset with a 5 km resolution (0.05° × 0.05°).  - From the anthropometric data, standardized indicators for height-for-age and weight-for-height z-scores were derived.  - The explanatory variables were mean temperature (in degrees Celsius) and precipitation (in millimeters). Annual means were calculated at the district level based on latitude and longitude. To account for long-term climate change, data were considered for two periods: 1981–2000 as the baseline period and 2007–2017 as the study period. The primary exposure was the calculation of mean variations in average temperature and precipitation values between these two periods. |
| Kamiya, 2025 [56] | Africa | Precipitation and Temperature | Yes | No | No | - The study utilized data from two Demographic and Health Surveys conducted in Mali in 2012–13 and 2018. Climate variables were obtained from the Advancing Research on Nutrition and Agriculture data, which integrates information from more than 4,000 weather stations.  - From the children's height and weight measurements, z-scores for anthropometric indices were calculated, identifying stunting (height-for-age), underweight (weight-for-age), and wasting (weight-for-height).  - Climate variables (precipitation and temperature) were obtained with a spatial resolution of 0.5° x 0.5°. Exposure was aggregated into periods of: 3 months, 6 months, 1 year, and 2 years prior to the surveys. Differences between the mean annual precipitation relative to the historical mean were also calculated, along with binary variables for dry climate (defined as annual precipitation below 200 millimeters). |
| Khan et al, 2020 [57] | Asia | Precipitation and Temperature | Yes | No | No | - The study utilized sociodemographic and anthropometric data of children from the Bangladesh Demographic and Health Surveys conducted in 2007, 2011, and 2014. Mean precipitation data per cluster were obtained using Climate Hazards Group InfraRed Precipitation with Station version 2.0 data, available every five years (1985 to 2015). Mean temperature data per cluster were obtained using WorldClim version 2 data, available for January through December of the survey year.  - The outcome variables used were the anthropometric characteristics of the children from the households included in the Bangladesh Demographic and Health Surveys.  - The climate explanatory variables were precipitation and temperature, measured by the mean annual values of the grid cells whose centroid was located within a 10 km radius (for rural locations) or a 2 km radius (for urban locations). Precipitation exposure was based on interpolated mean precipitation to calculate the specific value for the survey year (2007, 2011, and 2014), while the mean temperature data were available for the survey year itself. |
| LaPointe et al, 2024 [58] | Africa | Temperature | Yes | No | No | - A retrospective cohort study was conducted using data from 3,016 infants born to women interviewed as part of the impact evaluation of the Livelihood Empowerment Against Poverty program across five rural districts in northern Ghana between 2015 and 2017. A sample of program households and comparison households was assessed at baseline (2015, pre-intervention) and in 2017 (post-intervention). Mean weekly temperatures were obtained from the European Centre for Medium-Range Weather Forecasts Reanalysis v5 Land database.  - Birth weight was obtained through maternal report or recorded on a health card. Low birth weight was defined as a weight below 2.5 kilograms.  - The climate explanatory variable was the mean weekly temperature (measured in kelvin). The temporal exposure considered the 52 weeks prior to birth and was linked to household data via the centroids of the community coordinates. The analysis involved comparing program-participating households with comparison households, assessed at two time points (pre- and post-intervention). |
| Lazzaroni, Wagner, 2016 [59] | Africa | Drought and Cold wave | Yes | No | No | - A quantitative cross-sectional study with repeated sections was conducted, based on two rounds of a household panel survey carried out in rural areas of eight regions of Senegal in 2009 and 2011. The sample exclusively included farming households. Anthropometric data from children aged 12 to 60 months (n=1,694 in 2009 and n=2,116 in 2011), sociodemographic data, and self-reported shocks were used.  - The outcome indicator used was the weight-for-age z-score.  - The exposure was the occurrence of self-reported shocks (adverse events in the natural, biological, economic, and health spheres), including climate events such as drought and cold waves. In each survey round, respondents were asked whether the household had experienced a shock from a list of possible adverse events and the year the event occurred. |
| López-Carr et al, 2015 [60] | Africa | Precipitation | Yes | No | No | - A cross-sectional study was conducted using anthropometric data of children under 5 years of age from Demographic and Health Surveys (2008–2012) in the Lake Victoria Basin of East Africa (territorially managed by Tanzania, Kenya, and Uganda). Climate exposure data (precipitation) were obtained for the period from 1981 to 2012.  - The outcome variable was the proportion of children under 5 years of age with stunting within each survey cluster. The number of children with stunting was aggregated from the household to the cluster level. The cluster-level prevalence of stunting was converted into a continuous surface using ordinary kriging.  - The climate exposure analyzed was the monthly change in precipitation (in millimeters) over the 1981–2012 period. This change was calculated using Theil-Sen estimators, and the data were resampled to a 5 km resolution. The analysis involved the association of the resampled precipitation data with the continuous surface of stunting prevalence. |
| López-Carr et al, 2016 [61] | Africa | Precipitation | Yes | No | No | - A quantitative approach was employed, integrating anthropometric data (height-for-age) of children under 5 years of age, sourced from Demographic and Health Surveys (2008–2012), with monthly precipitation data (1981–2012) obtained through remote sensing from the Climate Hazards Group InfraRed Precipitation with Station dataset.  - The outcome used was the height-for-age of children under 5 years of age. Anthropometric information was aggregated into clusters and spatially interpolated using the ordinary kriging method, generating a continuous surface of child malnutrition prevalence.  - The explanatory variable was monthly precipitation (1981–2012), obtained by remote sensing at a spatial resolution of approximately 5 km. The analysis involved the association of the continuous surface of child malnutrition prevalence with the precipitation data. |
| MacVicar et al, 2017 [62] | Africa | Temperature and Precipitation | Yes | No | No | - A retrospective observational study was conducted based on hospital records. Data from 3,197 singleton births, occurring between 2012 and 2015 at the Bwindi Community Hospital in southwestern Uganda, were utilized. Daily precipitation data were estimated based on satellite observations using the Rainfall Estimator algorithm, version 2.0. Daily temperature data were obtained from the ERA-Interim reanalysis dataset from the European Center of Medium-range Weather Forecasts.  - The outcome variable used was birth weight.  - Daily precipitation and temperature data were used to compose the meteorological variables, which were aggregated by gestational trimester and validated using local meteorological data. |
| Mank et al, 2021 [63] | Africa | Precipitation | Yes | No | No | - A dynamic cohort study was conducted between 2017 and 2019. Sociodemographic, household characteristic, dietary, and anthropometric data were used from 1,439 children aged 7 to 60 months residing in households assessed by the Demographic and Health Surveillance System of Nouna, in rural northwestern Burkina Faso. Ground-based daily precipitation data were obtained from the National Meteorological Agency of Burkina Faso, based on daily measurements from 19 rain gauges in the vicinity of the Nouna area (> 80 km).  - From the anthropometric data, height-for-age and weight-for-height z-score indicators were derived; stunting and wasting were defined as values below -2 z-scores for these indicators.  - Daily precipitation was derived from a combination of ground and satellite observations. A stochastic resampling method was used to generate daily time series, applying a statistical correction to the database used. The 15 selected precipitation indicators were standardized into z-scores, representing the deviation of annual precipitation data for each study year (2016–2019) from the baseline precipitation data (1981–2019) per stratum. Reduced rank regression was applied as a dimension reduction technique to create a precipitation variability score, considering all 15 indicators. |
| McMahon & Gray, 2021 [64] | Asia | Precipitation and Temperature | Yes | No | No | - A cross-sectional study was conducted using secondary data from 222,572 children aged 24 to 59 months residing in municipalities included in 18 rounds of Demographic and Health Surveys, carried out between 1990 and 2018 in Bangladesh, India, Nepal, and Pakistan. The children's anthropometric and sociodemographic data were combined with high-resolution geospatial climate data (temperature and precipitation) obtained from the Climatic Research Unit and the Climate Hazards Group.  - From the anthropometric data, the height-for-age z-score indicator was derived, as well as the presence of stunting (below -2 z-scores) and severe stunting (below -3 z-scores).  - The climate explanatory variables were mean precipitation (in millimeters) and mean temperature (in degrees Celsius), standardized monthly anomalies for temperature and precipitation (in standard deviations, calculated by the difference between the mean and the historical average from 1983 to 2016), and the count of extreme days (hot, cold, dry, and rainy). Extreme days were defined based on percentiles (above the 95th percentile for hot or rainy days and below the 5th percentile for cold and dry days). Exposure was assessed across three critical periods: prenatal (9 months prior to birth), the first year of life, and the second year of life. |
| Merwe et al, 2022 [65] | Africa | Precipitation and Temperature | Yes | No | No | - A longitudinal panel study was conducted using data from three rounds of the Living Standards Measurement Study – Integrated Surveys on Agriculture, carried out in Nigeria during the periods of 2010–2011, 2012–2013, and 2015–2016. The sample included children under 5 years of age observed in two consecutive rounds. Precipitation and temperature data were obtained from the Climatic Research Unit dataset at the University of East Anglia.  - From the anthropometric data, indicators for stunting (low height-for-age) and underweight (low weight-for-age) were derived.  - The climate explanatory variables were precipitation and temperature. Climate data were aggregated into 5 km buffers around the georeferenced coordinates of the households. Exposure was measured by the variability of precipitation and temperature between the post-planting and post-harvest periods. For this purpose, monthly means for temperature and precipitation were obtained from July (post-planting) of the year prior to the survey until June (post-harvest) of the survey year. |
| Molina & Saldarriaga, 2017 [66] | South America | Temperature | Yes | No | No | - An observational panel study was conducted, combining climate time-series data with nutritional outcomes at birth. The database used includes the Demographic and Health Surveys from Bolivia, Colombia, and Peru (1990–2013) for anthropometric and sociodemographic data. Temperature time-series data were obtained from the "Terrestrial Air Temperature: 1900–2010 Gridded Monthly Time Series Version 3.01" dataset.  - The dependent variables included birth weight in grams, the occurrence of low birth weight (below 2,500 g), and the perception of small size at birth. Perception was reported by the mothers, with children being considered "small at birth" if their mothers reported they were small or very small at birth compared to other infants.  - For each municipality, the corresponding monthly temperature was assigned based on the nearest node to the municipality's centroid. Variability was defined as the standard deviation of the mean monthly temperature during the nine months prior to birth, relative to the historical mean (1950–2010) of the mother's municipality of residence. Temperature variability exposures were also evaluated considering the periods of 6 to 8 months, 3 to 5 months, and 0 to 2 months before birth. |
| Ngwira, 2020 [67] | Africa | Precipitation and Temperature | Yes | No | No | - Secondary data were utilized from 5,149 children residing in households included in the 2015 Malawi Demographic and Health Survey. Child registry data were combined with geographic location data through the cluster identification number of the survey households.  - From the anthropometric data, the variables for stunting (height-for-age below -2 z-scores), wasting (weight-for-height below -2 z-scores), and overweight (weight-for-height above +2 z-scores) were derived.  - The independent climate variables used included mean annual temperature and mean annual precipitation. Exposure was linked to the geographic location through the cluster identification number. |
| Nicholas et al, 2021 [68] | South America | Precipitation and Temperature | Yes | No | No | - A cross-sectional study was conducted based on data from 69,850 children aged 0 to 60 months residing in households included in the rounds of the Peru Demographic and Health Survey for the years 1996, 2000, 2004–2008, 2009, 2011, and 2012. Monthly climate data (temperature and precipitation) were obtained from the Climatic Research Unit Time Series database.  - The outcome was height-for-age z-score, derived from anthropometric data.  - The climate explanatory variables were transformed into standardized temperature and precipitation anomalies. These anomalies were calculated as 9-month moving averages and standardized relative to the local historical mean (1996–2012). Climate data were integrated with the survey data at the department level. The anomalies were assigned to the prenatal period and subsequent years of each child's life, with exposure assessed cumulatively and by specific age groups (anomalies during ages 0–1 for ages 1–2; anomalies during ages 0–2 for ages 2–3; anomalies during ages 0–3 for ages 3–4; and anomalies during ages 0–4 for ages 4–5). |
| Niles, Brown, 2017 [69] | Africa e Asia | Precipitation | No | Yes | No | - Precipitation data from the Climate Hazards Group InfraRed Precipitation with Station (covering the last 30 years) were combined with food security data from 2,095 smallholder farming households (low-income), included in household surveys from the Climate Change, Agriculture, and Food Security program. These data were collected between December 2010 and 2012 across 14 sites in 12 countries in West and East Africa and South Asia.  - Household food insecurity in the past year (measured in number of months, ranging from 0 to 12 months) was assessed using a questionnaire in which respondents indicated the months they experienced food shortages or struggled to feed their family.  - This precipitation anomaly was calculated based on the difference between the mean annual sum of the three most rainy cumulative months in the year prior to the survey and the 30-year climatological mean. The anomaly was categorized as: "Drier than average" (below one standard deviation from the mean), "Normal range" (within one standard deviation from the mean), and "Wetter than average" (above one standard deviation from the mean). |
| Omay et al, 2025 [70] | Africa | Precipitation | No | Yes | No | - Climate data were obtained from the Climate Hazards Group InfraRed Precipitation with Station version 2.0 dataset, which provides high-resolution precipitation measurements (daily and monthly, covering 1981 to 2023). Non-climate data (indicators such as population distribution, land factors, economic and political stability, and food trade metrics) were sourced from the Food and Agriculture Organization of the United Nations database.  - An indicator analysis was conducted to assess food security.  - The explanatory variables included precipitation, arable land, population, and economic stability. The Standardized Precipitation Index was used to evaluate the severity and duration of droughts across multiple time periods (3, 4, and 12 months). To analyze the relationships, the Pearson correlation coefficient was used to quantify the strength and direction of linear associations between the variables. |
| Paul et al, 2012 [71] | Asia | Cyclone | Yes |  | No | - A cross-sectional study was conducted based on primary data collected between October and November 2008 from 331 households in three coastal villages of Bangladesh (Angulkata, Tatulbaria, and Charkashem), which were severely affected by Cyclone Sidr (occurring in November 2007).  - Anthropometric indicators for women of reproductive age (body mass index) and for children aged 6 to 59 months (percentage of weight-for-height, percentage of height-for-age, and percentage of weight-for-age) were calculated and categorized to determine nutritional status classification.  - The primary exposure was Cyclone Sidr. Due to the absence of pre-Sidr data, the impact of the cyclone on nutritional status was obtained indirectly by comparing the difference between the nutritional indicator values reported in this study and the indicator values available from the Bangladesh Demographic and Health Survey, conducted between March 24, 2007, and August 11, 2007. |
| Petscavage et al, 2024 [72] | Africa | Droughts, floods, earthquakes, landslides, storms, and wildfires | Yes | No | No | - Cross-sectional demographic and health data (including height, weight, and hemoglobin) were utilized, sourced from probabilistic household samples of 51 rounds of Demographic and Health Surveys conducted in 30 countries across Sub-Saharan Africa between 2010 and 2019. Natural disaster events were collected from the global Emergency Events Database.  - The outcome variables were height-for-age and weight-for-height z-scores, calculated from anthropometric measurements.  - The primary exposure was the occurrence of natural disaster events (droughts, floods, earthquakes, landslides, storms, and wildfires), sourced from the Emergency Events Database and categorized according to the Disaster Risk Reduction Hazard Glossary. Children were considered exposed if at least one natural disaster event occurred in the municipality or equivalent administrative unit where they were located. The temporal exposure considered was the 3 to 12-month periods prior to the interview, with clusters located within a 5 to 10 km radius of the child’s home. |
| Rahut et al, 2024 [73] | Asia | Precipitation and Temperature | Yes | No | No | - Sociodemographic, climate variability, and anthropometric data were utilized from children aged 0 to 59 months residing in rural households included in the Demographic and Health Surveys of six South and Southeast Asian countries: India (2019–21), Bangladesh (2017–2018), Pakistan (2017–2018), Nepal (2016), Cambodia (2014), and Timor-Leste (2016).  - The outcome variables (stunting, underweight, and wasting—defined as a z-score < -2 for height-for-age, weight-for-age, and weight-for-height, respectively) were obtained from standardized indicators and aggregated at the cluster level.  - The climate explanatory variables were mean annual precipitation (2000 to 2020) and land surface temperature (specific years). The analysis was conducted at the cluster level or 5 km Primary Sampling Unit, with an exclusive focus on rural areas. |
| Randell et al, 2020 [74] | Africa | Precipitation and Temperature | Yes | No | No | - Data from 23,026 children aged 1 to 5 years residing in households included in four rounds of the Ethiopia Demographic and Health Survey (2000, 2005, 2011, and 2016) were combined with high-resolution climate data (temperature and precipitation) obtained via remote sensing. Precipitation data were sourced from the Climate Hazards Group InfraRed Precipitation with Station, and temperature data from the Climate Hazards Center InfraRed Temperature with Station.  - The outcome variables derived from anthropometric data were the height-for-age z-score and its respective classifications (stunting and severe stunting).  - The climate explanatory variables were the mean daily maximum temperature (in degrees Celsius) and the mean monthly precipitation (in centimeters). Climate data means were calculated within a 10 km buffer surrounding the household clusters. Exposure was comprehensively assessed by considering the means calculated during the prenatal period, in the child's early life (from birth until the survey date), and by evaluating temperature and precipitation means during each trimester in utero and for each season (Kiremt, Bega, and Belg) from birth until the month of the survey. |
| Randell et al, 2021 [75] | Asia | Precipitation | No | Yes | No | - Household data from 11,029 households included in the 2016 Nepal Demographic and Health Survey were linked (using the geographic coordinates of the household clusters) to data on earthquake shaking intensity and high-resolution precipitation data from the 2015 monsoon season. Total daily precipitation data were obtained from the Climate-Weather Research and Forecasting Model.  - A measure of household food insecurity was created based on the Household Food Insecurity Access Scale. Households were classified into four categories: food security, mild food insecurity, moderate food insecurity, and severe food insecurity, based on responses from household heads to a set of nine questions regarding the frequency of food shortages over the past 12 months.  - The explanatory variables included earthquake shaking intensity and the precipitation anomaly during the 2015 monsoon season. The precipitation anomaly was created by first calculating the mean total precipitation throughout the monsoon months (June to September) in each cluster for a 36-year reference period (1980 to 2015). Subsequently, z-scores were calculated for the 2015 monsoon precipitation based on deviations from the reference period. |
| Rodríguez-Llanes et al, 2011 [76] | Asia | Floods | Yes | No | No | - A population-based study with two-stage cluster sampling was conducted in September 2009 in the rural district of Jagatsinghpur, Odisha, India. The study included 122 villages affected and 143 villages unaffected by the floods in 2006 and/or 2008. Information was collected through face-to-face interviews with parents and anthropometric measurements of children aged 6 to 59 months.  - The outcome variables were standardized anthropometric indicators (height-for-age, weight-for-age, and weight-for-height z-scores), and their respective categorizations: stunting (height-for-age below -2 z-scores), severe stunting (height-for-age below -3 z-scores), underweight (weight-for-age below -2 z-scores), severe underweight (weight-for-age below -3 z-scores), wasting (weight-for-height below -2 z-scores), and severe wasting (weight-for-height below -3 z-scores).  - The exposure was flooding, assessed based on official data provided by the Odisha State Disaster Mitigation Authority. Exposure was categorized as: residing in an unaffected area, residing in an area affected in both 2006 and 2008, and residing in an area affected only in 2008.. |
| Rodriguez-Llanes et al, 2016 [77] | Asia | Floods | Yes | No | No | - A community-based cross-sectional study was conducted, featuring a village-stratified design and household sampling, in the Jagatsinghpur district of Orissa, India, shortly after the September 2008 floods. The household survey was carried out between October and November 2008, and the sample included 1,914 children from flooded villages and 1,614 from non-flooded villages (children aged 6 to 59 months).  - The outcome variables derived from anthropometric data were: stunting (height-for-age below -2 z-scores), underweight (weight-for-age below -2 z-scores), and wasting (weight-for-height below -2 z-scores).  - The exposure was flooding, defined as a binary variable (flooded/not flooded). Additional independent variables included household characteristics (income, religion, occupation, water source, food storage) and individual child characteristics (age, sex, birth weight, vaccination coverage). |
| Rojas Jr et al, 2023 [78] | Africa | Precipitation and Temperature | Yes | No | No | - Sociodemographic and anthropometric data were utilized from children aged 24 to 59 months residing in households in Burkina Faso, sourced from four rounds of the Demographic and Health Survey (conducted in 1993, 1998–1999, 2003, and 2010). Daily rainfall data from Climate Hazards Group InfraRed Precipitation with Station (from 1981 to 2010) and daily maximum temperature data from Climate Hazards Group InfraRed Temperature with Station (from 1983 to 2010) were used.  - The outcome variables were the anthropometric measurements of children aged 24 to 59 months.  - The explanatory variables were precipitation and maximum temperature. The time series of these data was linked to each household cluster location, considering a 10 km buffer. Standardized precipitation and temperature anomalies (in z-scores) were calculated using the difference between the annual mean and the long-term mean at the cluster level, divided by the long-term standard deviation. Seasonal anomalies were also calculated according to Burkina Faso's rainy and dry seasons, along with the number of local daily extremes for temperature and precipitation (number of hot, cold, wet, and dry days). These were defined as daily means greater than the 95th percentile or lower than the 5th percentile of values in a given cluster. The impact of early-life exposures (one year before birth, and the first and second years of life) to these variables was assessed. |
| Rosinger et al, 2023 [79] | South America | Floods | Yes | Yes | No | - A longitudinal panel study was conducted with two data collection points (featuring repeated measurements), carried out in two Tsimane communities in Beni, Bolivia. The first wave of collection occurred following the end of a flood (December 2013 to February 2014), and the second wave occurred approximately 8 weeks later. A total of 118 household heads and 129 children were interviewed.  - Outcome variables included the food insecurity score (measured with a nine-question version of the Household Food Security Survey Module, adapted for the previous month). Additionally, anthropometric data were collected for adults (weight, Body Mass Index, waist circumference, and the sum of skinfold thicknesses) and for children (weight-for-age, height-for-age, and Body Mass Index-for-age z-scores).  - The primary exposure was the flood, capturing the impact and recovery immediately after the extreme weather event. The analysis compared the two waves of data collection to assess changes in measurements following the event. The food insecurity measurement was adapted to focus on the preceding month. |
| Sam et al, 2019 [80] | Asia | Floods | No | Yes | No | - Household data were utilized from four villages (Beguniabasta, Gopalpur, Alanda, and Manijanga) in the Puri district of Odisha (rural India).  - Household food security was assessed through an index constructed based on 11 indicators organized according to the four dimensions established by the Food and Agriculture Organization of the United Nations (access, availability, utilization, and stability). These indicators were combined using polytomous principal component analysis.  - The primary exposure was the perception of flooding and environmental factors by household heads. The factors evaluated, relating to the period between 2009 and 2014, were: property losses due to flooding (durable goods, housing, livestock); presence of stress caused by flooding; mean precipitation variation (whether the family reported any variation); and income affected by environmental deterioration (due to climate change and flooding). |
| Sam et al, 2021 [81] | Asia | Drought | No | Yes | No | - Primary data were utilized, collected through a household survey with 157 rural families in the Balangir region of the state of Odisha, India, in areas highly vulnerable to drought.  - Household food security was assessed through an index constructed based on 11 indicators organized according to the four dimensions established by the Food and Agriculture Organization of the United Nations (access, availability, utilization, and stability). These indicators were combined using polytomous principal component analysis.  - The primary exposure was the perception of drought factors by household heads, relating to the period between 2009 and 2014. The factors evaluated included: perception of the impact of climate variations on agriculture (negative impact due to temperature and/or precipitation variation); drought-induced stress; unemployment due to drought; illness among family members due to drought; and the deterioration of the local income-generating environment due to drought. |
| Sandler, Sun, 2024 [82] | Africa | Precipitation and Temperature | Yes | No | No | - Sociodemographic and anthropometric data from children aged 0 to 59 months were combined (48,086 Nigerian children and 28,421 Kenyan children), residing in households included in the Demographic and Health Surveys of Kenya and Nigeria (2003, 2008–2009, 2013, 2014), with climate data. Climate data were obtained from the Climate Hazards Center InfraRed Temperature with Station.  - From the anthropometric data, the percentages of stunting (height-for-age below -2 z-scores) and wasting (weight-for-height below -2 z-scores) were calculated.  - Precipitation anomaly and temperature anomaly were continuous composite measures derived from the total mean monthly precipitation (in decimeters) and the mean monthly maximum temperature (in degrees Celsius) during the previous growing season. The precipitation anomaly was composed of the mean monthly precipitation anomaly relative to the mean of the last five years during the previous growing season. The temperature anomaly was composed of the mean monthly maximum temperature anomaly relative to the mean of the last five years during the previous growing season. The data were obtained at the cluster level of the households included in the surveys. |
| Scharadin et al, 2023 [83] | North America | Hurricane/tropical storm, wildfire, tornado, winter weather, severe storm/thunderstorm, lightning, coastal phenomena, floods, heatwave, and wind. | No | No | Yes | - Sociodemographic data and indicators of availability and accessibility from United States counties were utilized, sourced from the Farm Economy Branch of the Economic Research Service of the United States Department of Agriculture for the years 2010/2011 and 2015/2016. Data on extreme weather events were obtained from the Spatial Hazard Events and Losses Database.  - The outcome variables were measured at the county level and included: the density of specific food retail outlets (number of supermarkets, supercenters, and convenience stores per 100,000 inhabitants) as measures of availability; and the proportion of the low-income population with low access to supermarkets/supercenters and without a vehicle as a measure of accessibility.  - The exposure was the occurrence of extreme weather events at the county level between 2013 and 2017. The evaluated events were: hurricanes/tropical storms, wildfires, tornadoes, winter weather, severe storms/thunderstorms, lightning, coastal phenomena, flooding, heatwaves, and wind. |
| Silva et al, 2025 [84] | South America | Floods | No | No | Yes | - An ecological study was conducted in Rio Grande do Sul, Brazil, analyzing municipalities hit by the floods of May 2024. Geographic flood data extracted from Sentinel-2 satellites were used, along with information on commercial establishments obtained from the Federal Revenue Service's National Registry of Legal Entities for the year 2022.  - The outcome variables were the proportion of commercial establishments affected by classification group and the spatial distribution of impacts. Establishments were classified into three main groups based on the extent and purpose of food processing: unprocessed or minimally processed, ultra-processed, and mixed, with supermarkets and hypermarkets analyzed separately.  - The exposure was the geographic flood area. The interpolation of flood areas and georeferenced food retail establishments was performed using QGIS, with establishments located within the flood zone considered affected. Statistical analyses involved the calculation of frequencies, proportions, and the use of Pearson's chi-square test to compare impacts across categories. The spatial distribution of impacts was evaluated, along with an analysis of census tracts classified as food deserts (defined by a low density of healthy establishments), using Jenks Natural Breaks optimization maps. |
| Shaw et al, 2020 [85] | Asia | Drought | Yes | No | No | - An ecological cross-sectional study with spatial analysis was conducted, utilizing sociodemographic and anthropometric data from 259,627 children under 5 years of age, aggregated from 640 districts in India (which served as the units of analysis) during the 2015–2016 period. Information from the National Family Health Survey was integrated with climate indices derived from remote sensing.  - The outcome variables were anthropometric indicators (height-for-age, weight-for-height, and weight-for-age z-scores) and the nutritional status classification derived from these indicators: stunting (height-for-age below -2 z-scores), underweight (weight-for-age below -2 z-scores), and wasting (weight-for-height below -2 z-scores).  - Agricultural drought conditions were captured by the Scaled Drought Condition Index, which measures soil moisture deficit, precipitation, and temperature differences in arid and humid regions. Low values (near or equal to 0) indicate a severe drought condition, while in humid conditions, the value is near 1. The index was used to capture the predominant agricultural drought conditions in Indian districts during the study's reference period (2015–2016). |
| Shively, 2016 [86] | Asia and Africa | Precipitation and Temperature | Yes | No | No | - The study utilized data from 11,946 children under 5 years of age residing in households included in the 2006 and 2011 Demographic and Health Surveys conducted in Nepal (n=7,572) and Uganda (n=4,374). Precipitation data for Nepal were obtained from the Nepal Department of Hydrology and Meteorology, and for Uganda, from the Climate Hazards Group InfraRed Precipitation with Station. Gridded temperature data were sourced from the Berkeley Earth Project.  - From the anthropometric data, standardized indicators for height-for-age z-score and weight-for-height z-score were derived.  - The primary explanatory variables were standardized precipitation and temperature anomalies. Monthly precipitation (in millimeters) was standardized for each month and location based on a historical mean of 15 years (Nepal: 1998–2012) and 34 years (Uganda: 1981–2014), calculating the standardized monthly precipitation anomaly (deviations from the mean relative to the historical average). The temperature anomaly was calculated relative to a reference mean from January 1951 to December 1980. The independent standardized anomaly variables were constructed considering the following exposure periods: the gestational period, the year of birth, and the year the survey data were collected. |
| Skoufias & Vinha, 2012 [87] | North America | Precipitation and Temperature | Yes | No | No | - The study utilized data from children aged 12 to 47 months residing in rural areas of 169 municipalities in Mexico, included in the 1999 National Nutrition Survey. Precipitation and temperature data were obtained from the Mexican Institute of Water Technology.  - The standardized height-for-age indicator was derived from anthropometric data.  - Negative and positive precipitation and temperature anomalies were obtained from interpolated weather station data for a geographic centroid in each municipality. Total precipitation and accumulated growing degree-days—a cumulative temperature measure based on daily minimum and maximum temperatures—were calculated for each agricultural year (October to September) and for each rainy season (April to September). A negative anomaly was defined if the precipitation (or temperature) of the previous period was at least one standard deviation lower than the 1951–1985 mean. A positive anomaly was defined if the precipitation (or temperature) of the previous period was at least one standard deviation higher than the 1951–1985 mean. |
| Sultan et al, 2025 [88] | Africa | Precipitation and Temperature | Yes | No | No | - The study utilized data aggregated at the regional and national levels of children aged 6 to 59 months residing in Niger, sourced from the Standardized Monitoring and Assessment of Relief and Transitions (2005–2022), as well as Demographic and Health Surveys and Multiple Indicator Cluster Surveys (1992–2000). Climate data were extracted from satellite datasets such as Tropical Applications of Meteorology using Satellite and Climate Hazards Group InfraRed Precipitation with Station.  - The outcome variables were the prevalences of Global Acute Malnutrition and Severe Acute Malnutrition, considering the weight-for-height z-score.  - The explanatory variables included precipitation indices (accumulated precipitation from June to September; onset of rains; cessation of rains; length of the rainy season; and the number of dry spells) and temperature indices (mean land surface temperature; minimum temperature; maximum temperature; percentage of days with maximum temperature above the 90th percentile; percentage of days with minimum temperature above the 90th percentile; and the heatwave duration index). |
| Thiede, Gray, 2020 [89] | Asia | Precipitation and Temperature | Yes | No | No | - The study utilized anthropometric data from children under five years of age and household sociodemographic data from five waves of the Indonesian Family Life Survey (1993–1994, 1997–1998, 2000, 2007–2008, and 2014–2015). The database for temperature and precipitation variables was sourced from NASA’s Modern-Era Retrospective Analysis for Research and Applications.  - From the anthropometric data, height-for-age and weight-for-height were calculated.  - The climate explanatory variables included the mean delay in the onset of the monsoon, as well as the mean daily precipitation (in millimeters per day) and mean daily temperature (in degrees Celsius). The mean delay in the onset of the monsoon was calculated as the average number of days after August 1st until cumulative precipitation reached 20 mm. Mean daily precipitation and temperature were calculated over the specific periods analyzed: prenatal, 0 to 11 months of life, and 12 to 23 months of life (for height-for-age), and the last 12 months prior to the survey (for weight-for-height). |
| Thiede, Strube, 2020 [90] | Africa | Precipitation and Temperature | Yes | No | No | - The study utilized anthropometric and socioeconomic information from the Demographic and Health Survey across 18 countries in Sub-Saharan Africa (between 1990 and 2015), combined with monthly temperature and precipitation data from the Climatic Research Unit Time Series database.  - From the anthropometric information, the weight-for-height indicator was used.  - The climate explanatory variables (temperature and precipitation) were calculated as standard deviations relative to the local historical mean (1951–2015). Exposure was measured at the cluster level surrounding the households, considering the 12 months prior to data collection. |
| Tiwari et al, 2017 [91] | Asia | Precipitation | Yes | No | No | - The study utilized child- and household-level data obtained from three rounds of the Demographic and Health Survey conducted in Nepal in 2001, 2006, and 2011. The analysis focused on 13,683 children aged 0 to 60 months, born and residing in rural areas. Monthly precipitation data from a network of 171 rain gauge stations were obtained from the Nepal Department of Hydrology and Meteorology.  - The outcome variables derived from weight and height data were the standardized height-for-age z-score and weight-for-height z-score indicators.  - The monsoon rainfall anomaly (June to September) was obtained from precipitation data processed to yield, for each month, logarithmic deviations comparing current rainfall with the historical average (1972–1997). For the weight-for-height indicator, the impact of the monsoon rainfall anomaly in the current and previous seasons was estimated. For the height-for-age indicator, a cohort analysis was used to measure the effects of past rainfall in different years of the child's life, analyzing the accumulation of these impacts over time since birth. |
| Tusting et al, 2020 [92] | Africa | Temperature | Yes | No | No | - The study was a cross-sectional study utilizing secondary data from 52 Demographic and Health Surveys conducted between 2003 and 2016 across 29 countries in Sub-Saharan Africa. The sample included 656,107 children aged 0 to 5 years. Mean monthly daytime temperature data (measured between 2000 and 2016) were obtained and linked to the household clusters included in the survey.  - From the anthropometric data, indicators were derived for stunting (height-for-age < -2 z-scores), wasting (weight-for-height < -2 z-scores), concurrent stunting and wasting, and underweight (weight-for-age < -2 z-scores).  - The mean monthly daytime temperature was transformed into a categorical variable with the following intervals: < 30 °C, 30 °C to < 35 °C, and ≥ 35 °C. Exposure was linked to the household clusters included in the research. |
| Xiong et al, 2008 [93] | North America | Hurricane | Yes | No | No | - The study was a prospective cohort conducted between January 2006 and June 2007 in two locations in the United States: Tulane-Lakeside Hospital (New Orleans) and Woman's Hospital (Baton Rouge). The sample consisted of 301 newborns of women who were pregnant during Hurricane Katrina (August 2005) or became pregnant shortly after the event. Outcome data were obtained from medical records.  - The outcome variable was birth weight, with low birth weight defined as a weight of less than 2,500 grams.  - The primary exposure was exposure to Hurricane Katrina. High hurricane exposure was defined as having three or more severe experiences from a list of events (feeling that one's life was in danger, suffering illness or injury, walking in floodwaters, significant home damage, lack of electricity for more than a week, the death of someone close, or witnessing a death). Pregnant women were also interviewed during pregnancy regarding their experiences during the hurricane and the presence of symptoms of post-traumatic stress disorder and/or depression. |
| Yeboah et al, 2022 [94] | Africa | Precipitation | Yes | No | No | - The study utilized sociodemographic and anthropometric data from a household survey conducted in 2017, covering 12,919 children under 5 years of age in 6,890 households across 526 villages in 24 rural districts of Burkina Faso. Precipitation data were extracted via satellite by the Climate Hazards Group InfraRed Precipitation with Station.  - The height-for-age z-score was derived from the anthropometric data.  - Precipitation was obtained via monthly estimates at the household level (from September 2011 to June 2017), located at an average distance of 2 km from the cluster centroid. The primary exposure variable was "lifetime average precipitation exposure," calculated using the mean monthly cumulative precipitation from the start of gestation (assuming a 9-month pregnancy) until the month of the survey. |
| Yuan et al, 2025 [95] | Asia | Ambient temperature | Yes | No | No | - The study included data from 23,182 pregnant women (18 to 45 years old) with complete residence information and no pre-existing severe health conditions. Daily temperature and humidity data were obtained from 20 weather stations.  - The outcome variables were routine ultrasound measurements of fetal development parameters: head circumference, abdominal circumference, femur length, and estimated fetal weight. Measurements were standardized using z-scores adjusted for gestational age and sex.  - The primary exposure was heat stress, captured by apparent temperature, calculated using Thom’s Discomfort Index, which combines temperature and humidity. Apparent temperature was estimated by interpolating daily temperature and humidity data from 20 weather stations, linked to the participants' residential addresses. The temporal exposure was the mean apparent temperature calculated from the first day of the last menstrual period until each ultrasound examination. |

**Preferred Reporting Items for Systematic reviews and Meta-Analyses extension for Scoping Reviews (PRISMA-ScR) Checklist**

| **SECTION** | **ITEM** | **PRISMA-ScR CHECKLIST ITEM** | **REPORTED ON PAGE #** |
| --- | --- | --- | --- |
| **TITLE** | | | |
| Title | 1 | Identify the report as a scoping review. | Page 1 |
| **ABSTRACT** | | | |
| Structured summary | 2 | Provide a structured summary that includes(as applicable): background, objectives, eligibility criteria, sources of evidence, charting methods, results, and conclusions that relate to the review questions and objectives. | Page 1 |
| **INTRODUCTION** | | | |
| Rationale | 3 | Describe the rationale for the review in the context of what is already known. Explain why the review questions/objectives lend themselves to a scoping review approach. | Pages 1-2 |
| Objectives | 4 | Provide an explicit statement of the questions and objectives being addressed with reference to their key elements (e.g., population or participants, concepts, and context) or other relevant key elements used to conceptualize the review questions and/or objectives. | Page 2 |
| **METHODS** | | | |
| Protocol and registration | 5 | Indicate whether a review protocol exists; state if and where it can be accessed (e.g., a Web address); and if available, provide registration information, including the registration number. | Page 3 |
| Eligibility criteria | 6 | Specify characteristics of the sources of evidence used as eligibility criteria (e.g., years considered, language, and publication status), and provide a rationale. | Pages 4-5 |
| Information sources* | 7 | Describe all information sourcesin the search (e.g., databases with dates of coverage and contact with authors to identify additional sources), as well as the date the most recent search was executed. | Page 5 |
| Search | 8 | Present the full electronic search strategy for at least 1 database, including any limits used, such that it could be repeated. | Page 5 |
| Selection of sources of evidence† | 9 | State the process for selecting sources of evidence (i.e., screening and eligibility) included in the scoping review. | Pages 5-6 |
| Data charting process‡ | 10 | Describe the methods of charting data from the included sources of evidence (e.g., calibrated forms or forms that have been tested by the team before their use, and whether data charting was done independently or in duplicate) and any processes for obtaining and confirming data from investigators. | Page 6 |
| Data items | 11 | List and define all variables for which data were sought and any assumptions and simplifications made. | Pages 3,4, 6 |
| Critical appraisal of individual sources of evidence§ | 12 | If done, provide a rationale for conducting a critical appraisal of included sources of evidence; describe the methods used and how this information was used in any data synthesis (if appropriate). | not applicable |
| Synthesis of results | 13 | Describe the methods of handling and summarizing the data that were charted. | Page 6 |
| **RESULTS** | | | |
| Selection of sources of evidence | 14 | Give numbers of sources of evidence screened, assessed for eligibility, and included in the review, with reasons for exclusions at each stage, ideally using a flow diagram. | Pages 6-7 |
| Characteristics of sources of evidence | 15 | For each source of evidence, present characteristics for which data were charted and provide the citations. | Pages 7, 8-9, Table S2 |
| Critical appraisal within sources of evidence | 16 | If done, present data on critical appraisal of included sources of evidence (see item 12). | not applicable |
| Results of individual sources of evidence | 17 | For each included source of evidence, present the relevant data that were charted that relate to the review questions and objectives. | Pages 10-28 |
| Synthesis of results | 18 | Summarize and/or present the charting results as they relate to the review questions and objectives. | Pages 30-31 |
| **DISCUSSION** | | | |
| Summary of evidence | 19 | Summarize the main results (including an overview of concepts, themes, and types of evidence available), link to the review questions and objectives, and consider the relevance to key groups. | Pages 32-34 |
| Limitations | 20 | Discuss the limitations of the scoping review process. | Page 34 |
| Conclusions | 21 | Provide a general interpretation of the results with respect to the review questions and objectives, as well as potential implications and/or next steps. | Page 35 |
| **FUNDING** | | | |
| Funding | 22 | Describe sources of funding for the included sources of evidence, as well as sources of funding for the scoping review. Describe the role of the funders of the scoping review. | Page 35 |

JBI = Joanna Briggs Institute; PRISMA-ScR = Preferred Reporting Items for Systematic reviews and Meta-Analyses extension for Scoping Reviews.

* Where *sources of evidence* (see second footnote) are compiled from, such as bibliographic databases, social media platforms, and Web sites.

† A more inclusive/heterogeneous term used to account for the different types of evidence or data sources (e.g., quantitative and/or qualitative research, expert opinion, and policy documents) that may be eligible in a scoping review as opposed to only studies. This is not to be confused with *information sources* (see first footnote).

‡ The frameworks by Arksey and O’Malley (6) and Levac and colleagues (7) and the JBI guidance (4, 5) refer to the process of data extraction in a scoping review as data charting*.*

§The process of systematically examining research evidence to assess its validity, results, and relevance before using it to inform a decision. This term is used for items 12 and 19 instead of "risk of bias" (which is more applicable to systematic reviews of interventions) to include and acknowledge the various sources of evidence that may be used in a scoping review (e.g., quantitative and/or qualitative research, expert opinion, and policy document).

*From:*Tricco AC, Lillie E, Zarin W, O'Brien KK, Colquhoun H, Levac D, et al. PRISMA Extension for Scoping Reviews (PRISMAScR): Checklist and Explanation. Ann Intern Med. 2018;169:467–473.doi: 10.7326/M18-0850
